# Single-cell transcriptional dissection illuminates an evolution of immunosuppressive microenvironment during pancreatic ductal adenocarcinoma metastasis

**DOI:** 10.1038/s41392-025-02265-0

**Published:** 2025-06-09

**Authors:** Xiaowei Liu, Jinen Song, Meiling Yuan, Fengli Zuo, Huihui Li, Leyi Tang, Xinmin Wang, Xueyan Wang, Qian Xiao, Li Li, Xinyu Liu, Zhankun Yang, Jianlin Wu, Jing Jing, Xuelei Ma, Hubing Shi

**Affiliations:** 1 Institute for Breast Health Medicine, State Key Laboratory of Biotherapy, Frontiers Science Center for Disease-related Molecular Network, West China Hospital, Sichuan University, Chengdu, Sichuan China; 2 Department of Biotherapy, West China Hospital and State Key Laboratory of Biotherapy, Sichuan University, Chengdu, Sichuan China; 3 Institute of Clinical Pathology, West China Hospital of Sichuan University, Chengdu, China; 4 College of Chemical Engineering, Shijiazhuang University, Shijiazhuang, Hebei China; 5 State Key Laboratory of Quality Research in Chinese Medicine, Macau University of Science and Technology, Macau, China

**Keywords:** Cancer microenvironment, Metastasis, Tumour immunology

## Abstract

How the host immune system loses its surveillance function during the evolution from normal cell to malignancy is still largely unknown. Here, we investigate the dynamics changes of the pancreatic ductal adenocarcinoma (PDAC) tumor microenvironment by profiling 132,115 single-cell transcriptomes derived from 51 tissues, including healthy pancreatic tissue, non-metastatic PDAC primary tumors, metastatic primary tumors, and patient-matched liver metastases. The cellular proportion, bio-functional, and interaction between each cell type are carefully characterized. Aberrant copy number variations (CNVs) indicating malignant intensity are identified at chromosomes 7 and 20 of epithelial cells during tumor development. A bio-functional transition of predominant genes from physiology to pancreatic oncogenesis and metastasis is observed. Combinatorial analysis of epithelial cells and immunocytes indicates a gradient loss of immune surveillance during the malignant transformation. By dissecting cellular interaction, we unravel an incremental tumor cell-triggered apoptosis of DCs through molecular pair ANXA1-FPR1/3. Consequently, the activation and infiltration of cytotoxic CD8^+^ T cells are dampened progressively. Remarkably, we unveil a novel subtype of stress-response NK cells (strNK), which are characterized by robust proliferation, diminished cytolytic capabilities, and negative immune regulation. Notably, the presence of strNK cells is associated with poor prognosis of PDAC patients, implying a potential pro-tumor function. Taken together, our results not only shed light on the intricate mechanisms underlying step-wise evasion of immune surveillance during PDAC tumor development, but also provide a potential strategy for holding back malignant transition by reinforcing DCs’ function.

## Introduction

Pancreatic cancer remains one of the most formidable challenges in oncology, constituting approximately 8.5% of all cancer-related mortality.^1^ Pancreatic ductal adenocarcinoma (PDAC) is the most prevalent malignancy of the pancreas, accounting for around 80% of cases and with a 5-year survival rate below 12%.^2–4^ The therapeutic landscape for PDAC is complicated by the fact that a significant majority of patients, exceeding 80%, present with non-resectable disease and are characterized by regional or distant metastasis at diagnosis.^3^ Although surgical resection followed by adjuvant therapy is the standard treatment modality for those with localized, early-stage disease amenable to surgery, the ineffectiveness of chemotherapy for the non-resectable majority, underscores the urgent need for novel therapeutic strategies.^5,6^ Beyond the challenges of late detection and limited efficacy of conventional therapies, PDAC has proven resistant to immunotherapies such as immune checkpoint blockade, which has shown success in treating various other malignancies.^7–11^

The intricacies of the tumor microenvironment (TME) are the primary reason PDAC resists immunotherapy.^10,11^ Accumulating evidence highlights the correlation between higher numbers of neoantigens, robust anti-tumor CD8 T cell responses, and enhanced long-term survival, as well as the clinical efficacy of immune checkpoint blockade strategies.^12–15^ For the induction of specific adaptive immune responses against tumors, neoantigens derived from tumor cells are captured by antigen-presenting cells (APCs) and efficiently processed and cross-presented to CD8 T cells. However, dendritic cells (DCs), which are essential for the cross-presentation of antigens to CD8 T cells, exhibit markedly low infiltrated levels within the PDAC TME.^16,17^ Moreover, the immunosuppressive tumor-associated myeloid cells are also enriched in the TME of PDAC, further complicating the immune landscape.^12,18^ Collectively, these multifaceted elements contribute to the impediment of CD8 T cell infiltration, thereby maintaining the “immunologically cold” characteristics of the PDAC TME.

During the malignant transformation, tumor cells crosstalk with various stromal cell types within the TME, including T cells, natural killer (NK) cells, tumor-associated macrophages (TAM), and DCs, which are crucial for tumor progression and immune evasion. A comprehensive understanding of the complex interplay among the various cell types within the TME is essential for elucidating the mechanisms underlying tumor development, metastasis, and prognosis. Recent advancements in single-cell RNA sequencing (scRNA-seq) technology have enabled profiling the TME of PDAC primary tumors at the single-cell transcriptomic level.^12,19–21^ These studies have unveiled a considerable degree of heterogeneity in the immune and stromal composition of the TME across individual patients.^12,19,20^ Notably, the lack of T-cell infiltration has been associated with resistance to immunotherapy. However, current research, predominantly focusing on non-metastatic PDAC, falls short of comprehensively understanding the dynamic changes of the TME during the metastasis process. It is critical to acknowledge that PDAC exhibits a pronounced propensity for metastasis, and the dynamics of the TME undergo significant alterations during the metastatic process. Our previous studies revealed that there are distinct immune-tumor interactions in PDAC primary tumors, circulating tumor cells, and metastatic lesions.^22–24^ Consequently, there is a pressing need for comprehensive transcriptomic profiling of the TME at single-cell resolution across various stages of PDAC progression, including the process from healthy pancreatic tissue to non-metastatic primary tumors to metastatic primary tumors, and finally to form liver metastatic lesions, which is crucial for understanding the mechanism of PDAC metastasis and the development of more effective treatment paradigms.

In this study, we comprehensively dissect the TME landscape of healthy pancreatic tissue, non-metastatic PDAC primary tumors, metastatic primary tumors, and matched liver metastases. By leveraging the scRNA-seq, we characterize the variances of cell population distribution, cell transcriptomic profiles, and intercellular interactions throughout the malignant transformation of PDAC. We not only unravel the epithelial cell alteration during the evolution of PDAC metastasis but also characterize the transcriptomic landscapes of different immune cell subsets. Moreover, we identify several particular subtypes of immunocytes, including three types of macrophages, five types of DCs, ten types of T cells, and five types of NKs, that potentially contribute to the malignant transformation of the tumor. These results elucidate the complex interplay between various cellular constituents of the PDAC TME and their evolutionary dynamics during tumor progression.

## Results

### Transcriptionally profiling the cellular ecosystem during the process of PDAC metastasis

**Fig. 1:**
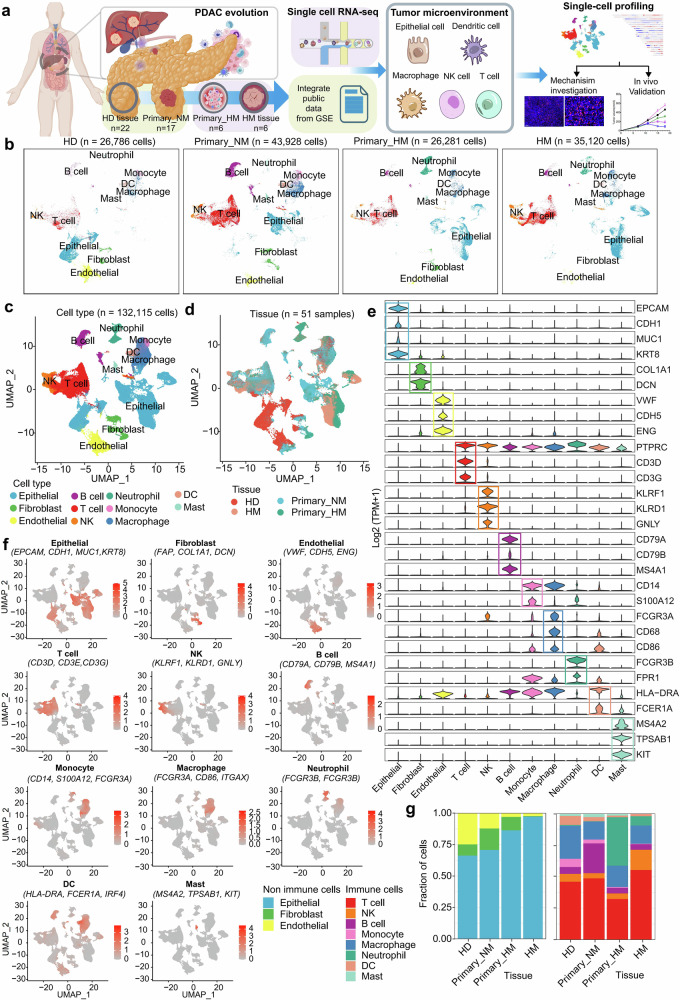
Characterization of the tumor microenvironment in PDAC metastasis. **a** Schematic representation of the experimental design aimed at dissecting the tumor microenvironment in PDAC metastasis. The image materials for (**a**) are sourced from the BioRender website. **b** UMAP plot shows the distinct cell types identified from biopsies of healthy donors (HD), primary tumors without metastasis (Primary_NM), primary tumors with metastasis (Primary_HM), and metastatic tumors (HM). The UMAP plots illustrate the distribution of all sequenced cells categorized by cell type (**c**) and tissue origin (**d**), with corresponding color codes indicated. **e** A violin plot displays the expression profiles of marker genes across different cell types. Colors represent the gene group affiliation. **f** The UMAP plot overlays the average expression levels of marker genes, scaled from gray to red, for the specified cell subtypes. Color intensity reflects the average logarithm of normalized expression data, with each dot representing an individual cell. **g** A bar plot quantifies the proportion of each cell type within the cohorts HD, Primary_NM, Primary_HM, and HM. The immune and non-immune cell fractions are calculated and presented separately

To dissect the evolution of tumor microenvironment during PDAC metastasis, we performed scRNA-seq on 6 liver metastatic patient-matched primary and metastatic lesions, 2 healthy donor pancreatic tissues (HD), and 2 primary non-metastatic pancreatic tumors (primary_NM) with 10× Genomics platform. Meanwhile, we integrate scRNA-seq data derived from two public data sets (CRA001160 and GSE205049), including 20 HD, and 15 primary_NM lesions (Fig. 1a). After quality control and batch effect correction (Supplementary Fig. 1a–d), total 132,115 single-cell transcriptome data are obtained, including 26,786 cells from 22 HD tissue, 43,928 cells from 17 primary_NM tissue, 26,281 cells from 6 primary tumor with hepatic metastasis (primary HM), and 35,120 cells from 6 hepatic metastasis (HM) lesions (Fig. 1b). Cell types are defined and presented by canonical markers, which are visualized by Uniform Manifold Approximation and Projection (UMAP) plots based on cell type, tissue origin (Fig. 1c, d), and patient origin (Supplementary Fig. 1e). *PTPRC* negative (CD45^*-*^) cells are further defined as epithelial cells (*EPCAM*^*+*^*, KRT8*^*+*^*, KRT18*^*+*^*, KRT19*^*+*^), endothelial cells (*VWF*^*+*^*, CDH5*^*+*^*, ENG*^*+*^*, PECAM1*^*+*^), fibroblast (*FAP*^*+*^*, COL1A1*^*+*^*, DCN*^*+*^), and *PTPRC* positive (CD45^*+*^) cells are further defined as B cells (*CD79A*^*+*^*, CD79B*^*+*^*, MS4A1*^*+*^), T cells (*CD3D*^*+*^*, CD3E*^*+*^*, CD3G*^*+*^), NKs (*KLRF1*^*+*^*, KLRD1*^*+*^*, GNLY*^*+*^), monocytes (*CD14*^*+*^*, S100A12*^*+*^), macrophages (*FCGR3A*^*+*^*, CD68*^*+*^*, CD86*^*+*^), neutrophils (*FCGR3B*^*+*^*, FPR1*^*+*^), dendritic cells (*HLA-DRA*^*+*^*, FCER1A*^*+*^), and mast cells (*MS4A2*^*+*^*, TPSAB1*^*+*^*, KIT*^*+*^) (Fig. 1e, f).

Our integrative analysis leveraged complementary strengths of three datasets: the CRA001160 cohort (predominantly contains non-immune cells from HD and Primary_NM samples), the GSE205049 dataset (contains immune cells from HD and Primary_NM samples), and our scRNA-seq data (encompasses both immune and non-immune cells across HD, Primary_NM, Primary_HM, and HM samples). Despite divergent experimental designs, systematic batch correction (RPCA, CCA, and Harmony) enabled robust integration of cell compartments across platforms, as evidenced by the seamless alignment of immune cells in low-dimensional UMAP projections (Supplementary Fig. 1f–l). In contrast, non-immune epithelial cells displayed clustering patterns that reflected their biological origins. Epithelial cells from HD and Primary_NM samples in both our dataset (P7 and P8, totaling 21,230 cells) and the CRA001160 dataset co-clustered (Supplementary Fig. 1h). However, epithelial cells from Primary_HM and HM lesions formed distinct clusters, indicating disease-specific heterogeneity in epithelial cells during the evolution of PDAC. These results confirm the reliability of data integration across datasets while revealing the disease-specific heterogeneity of epithelial cells.

The percentage and distribution of each cell type are presented by fractional column graphs (Fig. 1g and Supplementary Fig. 1m) and UMAP plots (Fig. 1c, d). Our integrated analysis demonstrates systematic identification of all major immune and stromal components across disease progression stages from healthy pancreas to metastatic lesions. These findings underscore the robustness of our data in capturing PDAC microenvironmental heterogeneity across disease stages. In addition, endothelial cells, which are derived from different patients and even organs, clustered together on the plot (Fig. 1c, d). It suggests that these nonmalignant cells, despite being hijacked by tumor cells from different patients (or organs), shared similar expression signatures. Moreover, we observed that immune and stromal populations from both HM and primary pancreatic lesions coalesced into transcriptionally coherent groups rather than segregating by tissue origin (Fig. 1c, d). This spatial convergence suggests pancreatic cancer cells may reprogram host microenvironments through dominant functional programs, effectively overriding hepatic niche signals to recapitulate pancreatic-like ecosystems. Critically, transcriptional overlap persisted even when excluding microenvironmental cells (Supplementary Fig. 1h), demonstrating inherent biological continuity between primary and metastatic epithelial compartments. These findings collectively validate HM as a sequential disease stage, where metastatic dissemination represents an evolved yet fundamentally continuous phase of pancreatic cancer progression. Taken together, these results suggest that the operating batch variation is limited and the foundational biology of hepatic metastases remains anchored in pancreatic cancer progression. Therefore, all the scRNA-seq data are qualified for further analysis.

### Characterization of epithelial cells alteration during the evolution of PDAC metastasis

**Fig. 2:**
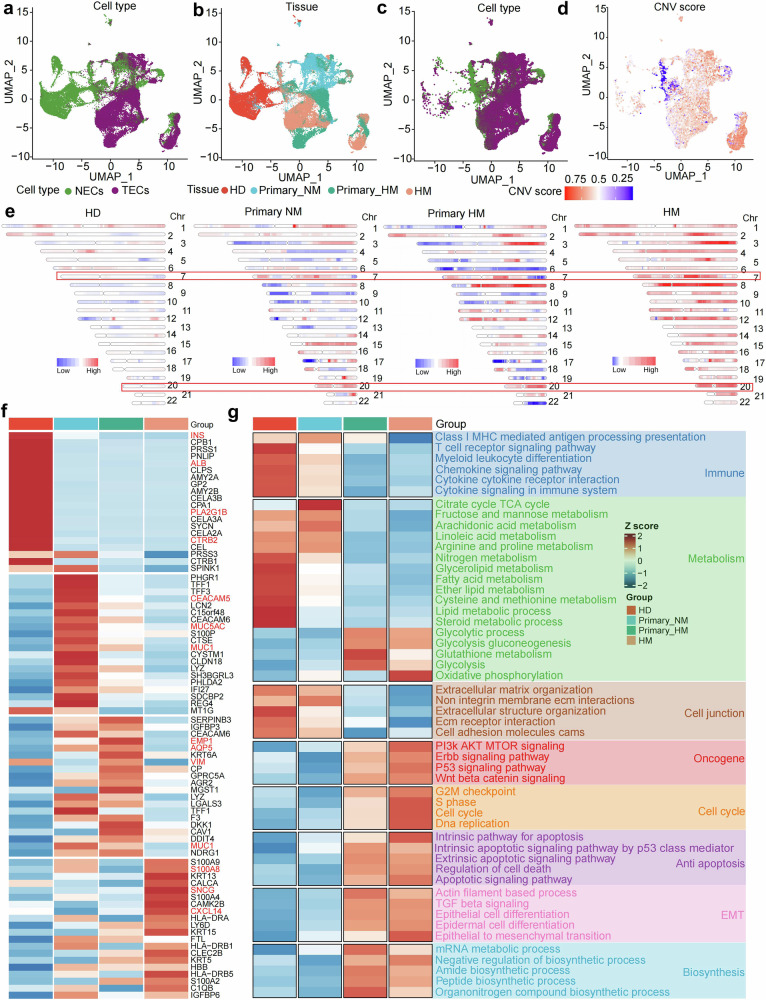
Analyze the alterations of epithelial cells during PDAC metastasis evolution. UMAP plots show the cell type (**a**) and tissue (**b**) origins of epithelial cells, with color codes provided at the base of each panel. **c** The UMAP plot highlights the tissue origin of epithelial cells isolated from Primary_NM, Primary_HM, and HM tissues. **d** The UMAP plot represents the CNV score of epithelial cells derived from Primary_NM, Primary_HM, and HM tissues. Details on the calculation of the CNV score are delineated in the methods section. **e** A chromosome map illustrates the CNV score distribution across different chromosomes for epithelial cells in HD, Primary_NM, Primary_HM, and HM samples. **f** The heatmap enumerates the top 20 DEGs for epithelial cells of each tissue origin. Color coding indicates the normalized z-score. **g** The heatmap shows the differentially enriched gene sets between epithelial cells of distinct tissues. The gene sets are categorized as follows: immunology (navy blue), metabolism (green), cell junction (maroon), oncogene (red), cell cycle (orange), anti-apoptosis (purple), EMT (magenta), and biosynthesis (cyan)

An interesting phenomenon in the percentage results of each cell type is that the proportion of epithelial cells gradually increases from healthy tissue to metastatic lesions (Fig. 1g). To unravel the mechanism underlying it, we first focus on the epithelial population. The normal epithelial cells (NECs) and tumor malignant epithelial cells (TECs) are discriminated by malignancy index CNV with CopyKT algorithm (Supplementary Fig. 2a). The transcriptomes of epithelial cells are plotted by UMAP based on malignant intensity, tissue origin, and CNV score (Fig. 2a–d). Interestingly, although the normal epithelial cells and malignant tumor cells show obvious differences in gene copy number alteration, the CNV score between primary tumor cells without metastasis, primary tumor cells with metastasis, and metastatic tumor cells are comparable (Fig. 2b, d). In another word, it is very hard to characterize the intensity of aberrance by overall CNV score. Then we dissect the CNV of each chromosome in malignant tumor cells from biopsies mentioned above. The aberrant CNVs are observed in certain chromosomes, which are well accepted as standard for judgement of malignant tumors, e.g. chromosome 3, 7, 8, 9, and 20 (Fig. 2e). Remarkably, in terms of characterization of malignant intensity during tumor evolution, the CNVs of chromosome 7 and 20 seem to be much more reliable and consistent, suggesting a potential function of the diagnostic target (Supplementary Fig. 2b).

Subsequently, we attempt to characterize the alteration between each stage at gene level (Fig. 2f and Supplementary Fig. 2c). The differential gene expression analysis indicates that the genes related to physiological function of pancreases, such as *INS, ALB, CTRB2*, and *PLA2G1B* are highly expressed in epithelial cells from healthy tissue. In the primary tumor without and with metastasis, genes related to pancreatic oncogenesis (such as *CECAM5*, *MUC1*, and *MUC5AC*)^25^ and metastasis (such as *EMP1, AQP5*, and *VIM*)^26^ are enriched, respectively. Genes potentially promoting metastasis are identified in tumor cells from metastatic lesions, including *S100A8*, *SMCG*, and *CXCL14*.^27^ We summarize the biofunction of these differential expressed genes by gene set enrichment analysis (GSVA), which enriches into eight categories of signature (Fig. 2g). The signatures related to lipid metabolism, cell-cell junction, and immune surveillance are enriched in normal pancreatic epithelial cells, and decrease gradually from benign organ to malignant tissues. The signatures of oncogene pathways, cell cycle, anti-apoptosis, glycolysis, epithelial-mesenchymal transition, and bio-macromolecule synthesis increase along with the tumor malignant evolution. Notably, the decrease of immune signatures related to lymphocytes and myeloid cells highlights the dysfunction of immune surveillance system during the tumor evolution. Additionally, metastatic tumor cells exhibited immune regulatory characteristics similar to those of their primary tumor counterparts, suggesting that the immune microenvironment in metastatic sites is shaped by tumor cells originating from the primary tumor (Fig. 2g). Given the dynamic evolution of tumor cells during PDAC progression and metastasis, we next aimed to investigate the functional alterations in immune cell subtypes concomitant with tumor cell evolution.

### Alteration of macrophages during the evolution of PDAC metastasis

Total 19,661 myeloid cells are identified and presented by UMAP plots. Macrophage (9970 cells), DCs (1812 cells), neutrophil (5622 cells), mast cells (1074 cells), and monocytes (1383 cells) are defined by indicated canonical markers mentioned above (Supplementary Fig. 3a–d). Macrophages and DCs are observed abundantly among all three datasets from each tissue origin. Monocytes are predominantly found in biopsys of HD and primary tumors without metastasis, and mast cells are barely observed. (Supplementary Fig. 3e).

**Fig. 3:**
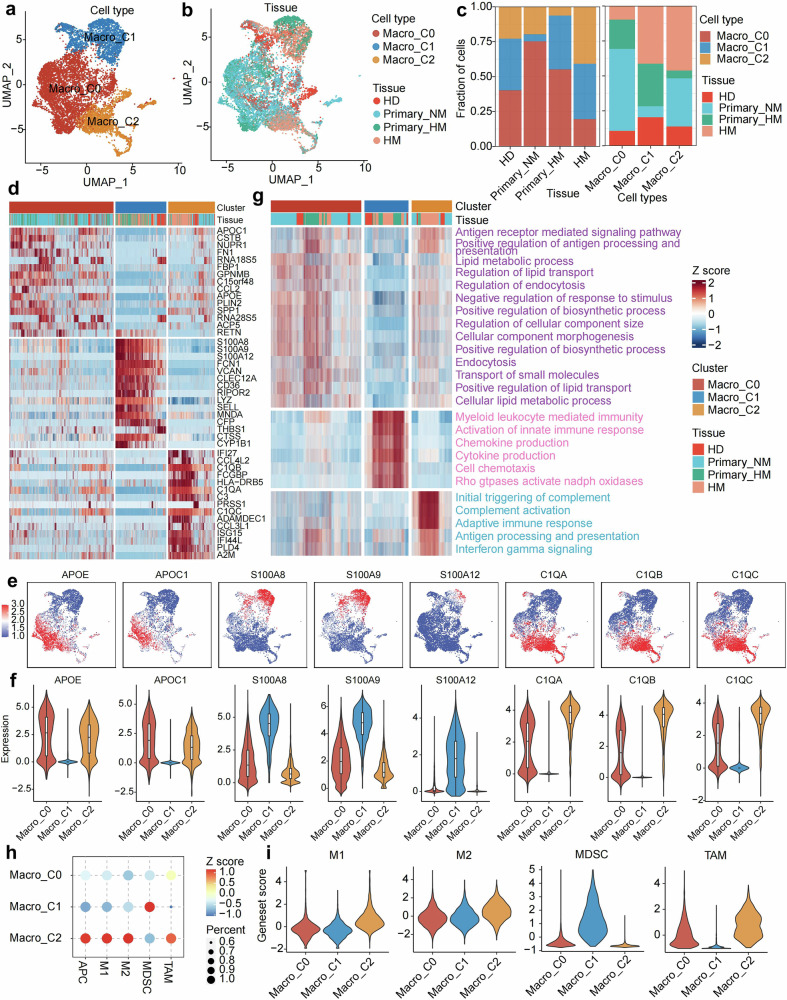
Alteration of macrophages during the evolution of PDAC metastasis. **a** UMAP projection illustrates the sub-clustered macrophages derived from all biopsies, with cells distinguished by color according to their cell type. **b** A UMAP plot delineates the tissue origin of the macrophages. **c** A bar plot quantifies the proportion of each macrophage subtype within the cohorts HD, Primary_NM, Primary_HM, and HM. **d** The top 15 differentially expressed genes for each macrophage subtype are displayed, with color representing the normalized z-score. **e** The UMAP plot projects the expression of marker genes for identified macrophage subtypes, ranging from blue to red, with three distinct clusters characterized by specific gene expression: macro C0 (*APOE*^+^, *APOC1*^+^), macro C1 (*S100A8*^+^, *S100A9*^+^, *S100A12*^+^), and macro C2 (*C1QA*^+^, *C1QB*^+^, *C1QC*^+^). **f** Violin plots depict the expression profiles of marker genes for each macrophage subtype, showing the variability in gene expression within each cluster. **g** The differentially enriched gene sets for each macrophage subtype are presented, with distinct colors for macro C0 (purple), macro C1 (magenta), and macro C2 (cyan). **h** A bubble heatmap represents the scores of canonical signatures corresponding to each cell type, where color reflects the z-score of the geneset score, and dot size indicates the percentage of signature-positive cells. **i** Violin plots display the canonical macrophage characters across each subtype cell

Macrophages are further categorized into three populations based on unsupervised clustering (Fig. 3a). C0 shows a non-metastatic primary lesion preferred pattern (Fig. 3b, c). Lipid metabolism related genes, such as *APOE* and *APOC1*, are up-regulated in C0 (Fig. 3d–f). This character is confirmed by GSVA signature analysis (Fig. 3g). Thus, we defined this population as early-stage tumor associated macrophage (esTAM). MDSC marker genes (*S100A8*, *S100A9*, and *S100A12*) are observed as the representative differential expressed genes of C1 (Fig. 3d–f). The signatures of chemotaxis and cytokine production are enriched in this population (Fig. 3g). Consistently, the MDSC signature score is highlighted in this population, which can be defined as MDSC like macrophage (Fig. 3h, i). Notably, the C1 population shows a metastatic primary lesion preferred pattern (Fig. 3c), implying a pro-metastasis function. The complement related genes (*C1QA*, *C1QB*, and *C1QC*) are observed in the C2 population (Fig. 3d–f), which also pinpoints the canonical TAM (cTAM) signature (Fig. 3h, i).

Moreover, we analyzed the characteristics of neutrophils during PDAC evolution. Neutrophils across different tissue origins were isolated and clustered for subsequent DEG and functional enrichment analyses (Supplementary Fig. 3f, g). Our integrative analysis revealed a dynamic functional evolution of neutrophils during pancreatic cancer progression. In HD tissues, neutrophils maintained homeostatic maintenance functions characterized by extracellular matrix organization and digestive enzyme regulation (Supplementary Fig. 3h). Upon transitioning to Primary_NM tumors, these cells acquired pro-proliferative capacities alongside adaptive immune modulation capabilities (Supplementary Fig. 3i). Notably, neutrophils within Primary_HM tumors demonstrated enhanced inflammatory activation and apoptosis-priming phenotypes (Supplementary Fig. 3j). Strikingly, HM neutrophils underwent profound immunosuppressive reprogramming through IL-10 signaling activation and chemotactic dominance mechanisms (Supplementary Fig. 3k). These stage-specific adaptations highlight neutrophils’ functional plasticity in shaping tumor permissiveness, suggesting microenvironment-driven reprogramming as a critical driver of metastatic competence.

### Evolution of PDAC metastasis accompanies the function loss of DC

**Fig. 4:**
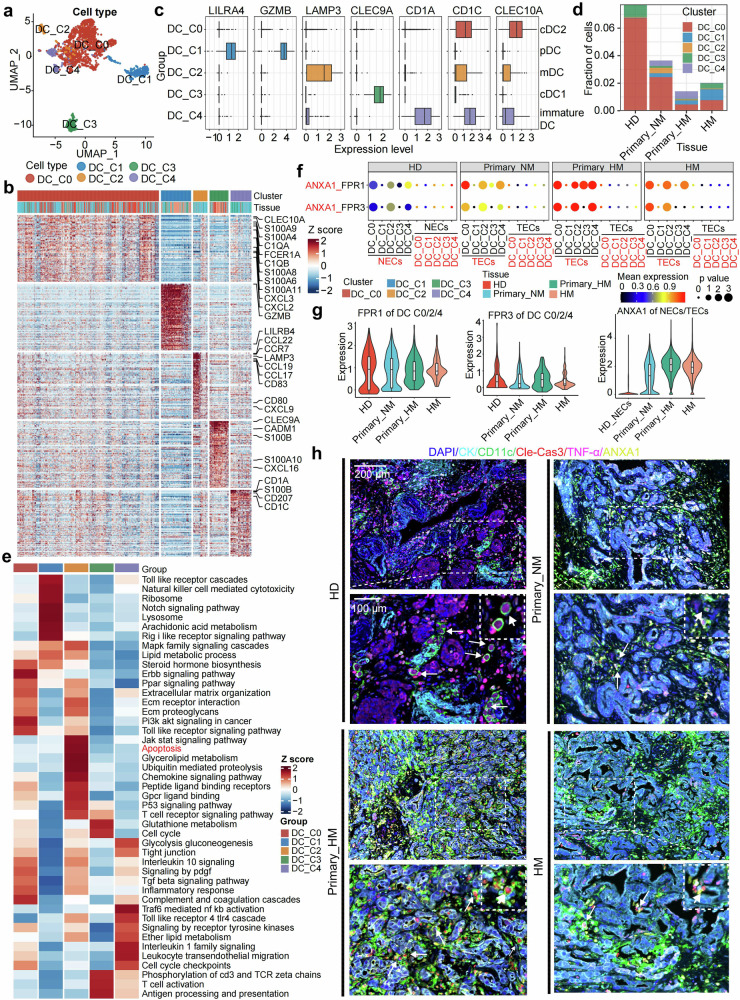
Evolution of PDAC metastasis accompanies the function loss of DC. **a** UMAP plot represents the sub-clustered DCs from all biopsies, with cells color-coded according to cell type for clear differentiation. **b** A heatmap shows the DEGs for each DC subtype, with representative marker genes highlighted and color indicating the normalized z-score. **c** A box plot illustrates the expression of marker genes across different cell types, including pDC (*LILRA4*^+^, *GZMB*^+^), mDC (*LAMP3*^+^), cDC1 (*CLEC9A*^+^), cDC2 (*CD1C*^+^, *CLEC10A*^+^), and immature DC (*CD1A*^+^), with color denoting gene group affiliation. **d** A bar plot displays the proportion of each DC subtype within the immune cell population. **e** A heatmap represents the differentially enriched gene sets for each DC subtype. **f** The dot plot illustrates the expression levels of ligand-receptor pairs, specifically ANXA1_FPR1/2, between TECs/NECs and DC subtypes across tissues. Color intensity reflects the mean expression of the ligand and receptor genes, while dot size corresponds to the statistical significance of the interactive molecular pairs. **g** Violin plots present the expression levels of *FPR1* on cDCs across different tissues (left), *FPR3* on cDCs (middle), and *ANXA1* on TECs/NECs (right). **h** Representative images of mIHC staining with HD tissue, primary_NM tumor, primary_HM tumor, and HM lesion. DAPI (blue), CD11c (green), CK (cyan), cleaved-caspase3 (red), TNF-α (purple), ANXA1 (yellow). Scale bars, 200 μm, or 100 μm

Total 1812 DC cells are identified from each tissue mentioned above and spontaneously clustered into 5 groups on UMAP plots (Fig. 4a and Supplementary Fig. 4a). The representative genes are characterized by differential gene expression analysis, followed by signature score enrichment analysis. Overall, the populations of C0, C2, C3, and C4 show a strong character of conventional dendritic cells (cDCs), while the C1 group shows a character of plasmacytoid dendritic cells (pDCs) (Supplementary Fig. 4b, c). The detailed digestion of representative genes shows that genes related to type 2 conventional dendritic cells (cDC2), mature DC (mDC), type 1 conventional dendritic cells (cDC1), and immature DC are specifically upregulated in C0 (*CLEC10A, FCGR2B*), C2 (*CCL22, LAMP3, CCR7, CD80, CD83*), C3 (*CLEC9A, CADM1, CXR1*), and C4 (*CD1A, CD207*), respectively (Fig. 4b, c, Supplementary Fig. 4d). Whereas, the pDC related genes, such as *LIRA4, GZMB*, are upregulated in C1. This cell definition is confirmed by the analysis of canonical maker genes for DC subtypes (Fig. 4c).

When we plot the proportions of each DC subgroup in total immunocytes (CD45^+^), we notice a remarkable decrease in cDC2 population (C0) during tumor evolution (Fig. 4d). To unravel the mechanism underlying the loss of this functional population, we explore the development hierarchy of these DCs by two independent algorithms, namely Velocyto and Monocle3 (Supplementary Fig. 4e, f). Both of them show a trajectory from immature DC to cDC2 to mature DC, which implies the road map of their life span. This indicates that mature DCs (C2) may represent a terminal state in the developmental hierarchy of these cells. Actually, the apoptosis related signature is highly enriched in the C2 cluster compared with the others (Fig. 4e). We then focus on the reason why the mDC undergoes cell apoptosis during the tumor evolution. The molecular interactions between each subtype of DCs and epithelial cells from benign to malignant tissues are profiled by CellPhoneDB (Supplementary Fig. 4g). The results demonstrated that the ANXA1:FPR1/FPR3 interaction exhibited the most significant and gradual increase during the process of malignancy transition (Fig. 4f). The expressional dissection indicates that ANXA1 on epithelial cells, rather than FPR1/3 on DCs, gradually increase from HD to non-metastatic primary tumor to metastatic primary tumor (Fig. 4g). Notably, high ANXA1 expression was significantly associated with worse overall survival in the TCGA PDAC cohort (Supplementary Fig. 4h), further supporting its potential role as a prognostic biomarker in pancreatic cancer. In addition, FPR1/3 predominantly distributes on C0, C2, and C4. Among these clusters, C0 presents the highest level of FPR1/3, which partially addresses the decrease of the population during tumor evolution (Supplementary Fig. 4i). To validate the apoptosis of DCs, we performed multiplex immunohistochemistry (mIHC) staining on biopsies of HD, non-metastatic primary tumor, metastatic primary tumor, and HM lesion. The results indicate that during tumor progression, the density of DCs (CD11c^+^) decreases, and they experience progressive apoptosis, as evidenced by cleaved caspase 3 (Fig. 4h and supplementary Figs. 4j, k). This process is accompanied by the increase of ANXA1 and decrease of TNF-α in epithelial cells (CK^+^) and DCs, respectively. According to previous report, the down-regulation of TNF-α usually indicates the anergy of DCs.^28^ All these data pinpoint the lost of function and shrinkage of population of DCs under the influence of tumor cells during the process of malignant progress.

**Fig. 5:**
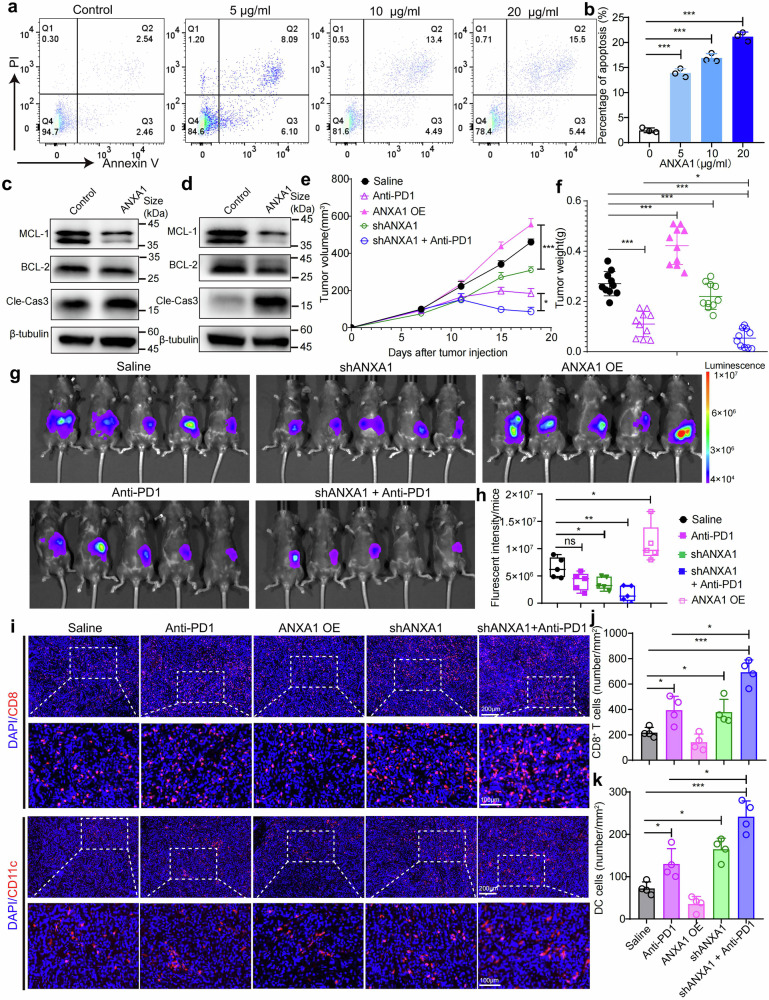
Validation of ANXA1 function in vitro and in vivo. The apoptosis of BMDC cells treated with indicated doses (0, 5, 10, 20 μg/ml) of ANXA1 was evaluated by Annexin V-FITC/PI based flow cytometry (**a**) and the results were quantified (**b**). Proteins indicative of apoptosis pathway activity, including MCL-1, BCL-2, and cleaved caspase3, were detected in BMDC (**c**) and DC2.4 (**d**) cells treated with 10 μg/ml ANXA1 for 24 h. **e**, **f** The PDAC xenograft model was established by subcutaneous injection of KPC cells with or without manipulation of the expression of ANXA1. Anti-PD1 treatment started when tumor volume achieved ≈80 mm^3^. The average tumor growth curves were profiled during treatment (**d**), and the tumor weight was recorded at the end of the treatment (**e**). **g**, **h** Mouse PDAC orthotopic models were established by injecting luciferase-tagged KPC cells (KPC-Luc) with ANXA1 overexpression, ANXA1 knockdown, or empty vector into the pancreas tissues of mice. Anti-PD-1 treatment (2.5 mg/kg, i.v.) was administered every 6 days after 5 days of the tumor inoculation. The tumor growth was visualized (**g**) and quantified (**h**) by bioluminescence imaging system 20 days after tumor inoculation. **i** Whole slide immunofluorescence staining of CD8 and CD11c to assess the infiltration of T cells and DCs in subcutaneous PDAC tumors. Scale bar 200 μm or 50 μm. **j**, **k** Quantification of the densities of CD8^+^ T cells (**h**) and CD11c^+^ DCs (**i**) in each group from (**i**)

Inspired by this observation, we wonder whether ANXA1 can induce the apoptosis of DCs and influence the efficiency of immunotherapy on PDAC. Firstly, we treated the activated bone marrow-derived DCs (BMDCs) with varying doses of ANXA1 protein and observed a significant induction of apoptosis in vitro (Fig. 5a, b). The results were further confirmed by the western blot (Fig. 5c, d, and Supplementary Fig. 5a, b). The results showed that ANXA1 effectively upregulates the expression of apoptotic marker cleaved caspase-3 while downregulating the anti-apoptotic marker MCL-1 and BCL-2. These results indicate that ANXA1 induces the apoptosis of DCs.

To further validate the in vivo function of ANXA1, we established both subcutaneous and orthotopic mouse PDAC models by implanting KPC cells with or without manipulation of ANXA1 expression, followed by treatment with anti-PD-1 blockade antibody. In the subcutaneous model, tumor growth curves were plotted during treatment, and the tumor weight was recorded at the end of the treatment (Fig. 5e, f). The results demonstrated that knockdown of ANXA1 exhibited potent anti-tumor efficacy, which was further enhanced by anti-PD-1 blockade antibody. Conversely, overexpression of ANXA1 slightly exacerbated tumor growth. Consistently, the orthotopic mouse PDAC model also confirmed that ANXA1 knockdown significantly inhibited tumor growth, while ANXA1 overexpression promoted tumor progression (Fig. 5g, h). Additionally, ANXA1 knockdown efficiently enhances the antitumor efficiency of PD-1 blockade on the orthotopic mouse PDAC model.

To determine whether manipulating the expression of ANXA1 will regulate the infiltration of cytotoxic T cells and DCs, we visualized CD8^+^ T cells and CD11c^+^ DCs by immunofluorescence (Fig. 5i–k). Consistent with the results of the tumor curve, quantitative analysis of whole slide imaging revealed that ANXA1 overexpression led to a decrease in the density of both CD8^+^ T cells and CD11c^+^ DCs. Conversely, both ANXA1 knockdown and anti-PD-1 treatment significantly increased the density of CD8^+^ T cells and CD11c^+^ DCs. Notably, PD-1 blockade further augmented the density of CD8^+^ T cells and CD11c^+^ DCs. The phenomenon was further validated by multiparameter flow cytometry analysis (Supplementary Fig. 5c–l). Consistently, CD3^+^ T cells, CD8^+^ cytotoxic T cells (CTLs, CD3^+^CD8^+^), total DCs (CD45^+^CD11c^+^), and mature DCs (CD11c^+^CD80^+^) in tumors were stimulated by ANXA1 knockdown, which was further enhanced by additional PD-1 blockade. On the contrary, ANXA1 overexpression led to a significant decrease in the infiltration of these immune cell populations. Interestingly, we found that changes in ANXA1 expression did not significantly alter the expression levels of these T cell exhaustion markers, such as PD1, TIM3, and CTLA4 (Supplementary Fig. 6).

In the orthotopic model, flow cytometry and immunofluorescence staining further validated these results. Flow cytometry showed that ANXA1 knockdown and anti-PD-1 treatment significantly enhanced the infiltration of CD3^+^ T cells, CD8^+^ cytotoxic T cells, CD11c^+^ total DCs, and CD11c^+^CD80^+^ mature DCs, while ANXA1 overexpression decreases their infiltration (Supplementary Fig. 7a–j). Notably, PD-1 blockade further augmented the infiltration of CD8^+^ T cells and CD11c^+^ DCs, suggesting that anti-PD-1 treatment and ANXA1 knockdown synergistically promote the anti-tumor immune response. The phenomenon was further validated by the immunofluorescent staining of CD8 and CD11c (Supplementary Fig. 7k–m). In addition, NK cell infiltration was not significantly affected by ANXA1 modulation in either model. Taken together, the number of tumor-infiltrating CD8^+^ T cells was positively correlated with the density of DCs, indicating that ANXA1-induced DC dysfunction and subsequent decrease in DC numbers may impede the anti-tumor immune response.

### Downregulation of cytotoxic lymphocytes during PDAC evolution

**Fig. 6:**
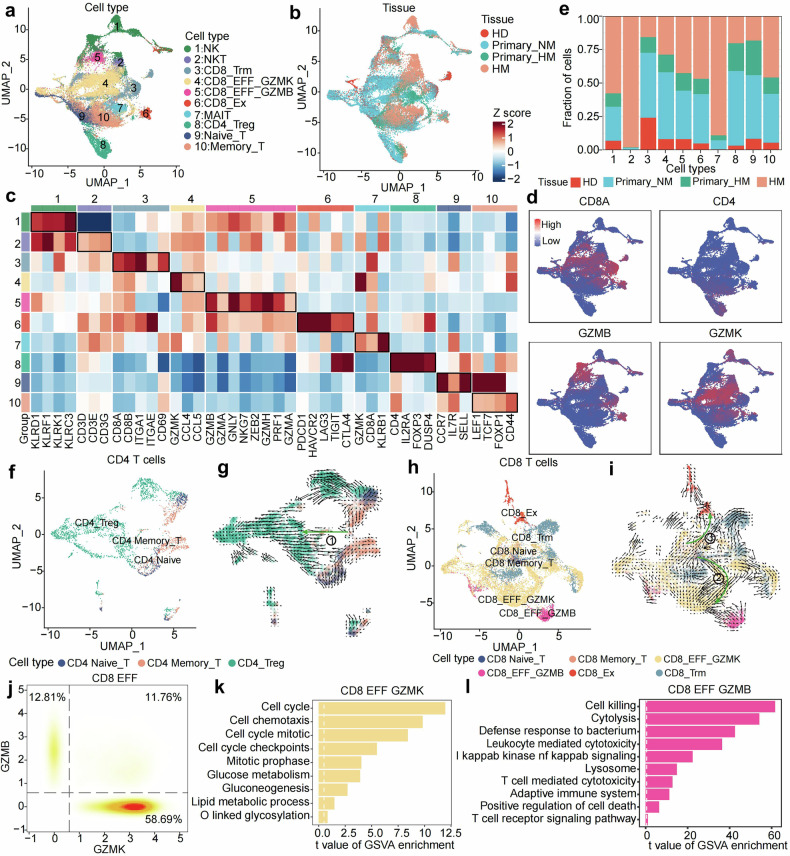
Downregulation of cytotoxic lymphocytes during PDAC evolution. **a** UMAP plot delineates the sub-clustered T lymphocytes and NK cells from all biopsies, with cells color-coded according to cell type for clear distinction. **b** The UMAP plot illustrates the tissue origin of T lymphocytes and NK cells. **c** A heatmap displays the expression patterns of marker genes characteristic of each cell type. **d** The UMAP plot projects the expression of marker genes for indicated cell subtypes, color-coded from blue to red. **e** A bar plot quantifies the proportion of T cell subtypes and NK cells within the cohorts HD, Primary_NM, Primary_HM, and HM. **f** UMAP projection illustrating the spatial distribution of CD4^+^ T cells across HD, Primary_NM, and Primary_HM groups. **g** Cell differentiation trajectories among CD4^+^ T cell subtypes in HD, Primary_NM, and Primary_HM, visualized through Velocyto analysis, with distinct cell populations color-coded by their respective phenotypes. **h** UMAP visualization demonstrating the spatial organization of CD8^+^ T cells in HD, Primary_NM, and Primary_HM samples. **i** Velocyto-based trajectory mapping of CD8^+^ T cell subtype differentiation in HD, Primary_NM, and Primary_HM, with cellular identity indicated by color-coded annotations. **j** Density plot depicts the bimodal distribution of GZMK and GZMB expression, with the percentage of each group labeled in the corners and color representing cell density. **k** A bar plot illustrates the t values of enriched gene sets for CD8 EFF GZMK cells, indicating the significance of gene expression changes. **l** A bar plot shows the t values of enriched gene sets for CD8 EFF GZMB cells, providing a measure of differential expression

Theoretically, cytotoxic lymphocytes cannot be fully activated without the support of DCs.^29^ We then profile and dissect the T lymphocytes and NKs by UMAP plot (Fig. 6a, b and Supplementary Fig. 8a, b). Total 33, 059 cells are presented and defined as CD8 Trm (*ITGA1*^*+*^*, ITGAE*^*+*^), mucosal associated invariant T (MAIT) cells (*KLRB1*^*+*^*, IL7R*^*+*^), GZMK CD8 effector (EFF) (*GZMK*^*+*^*, CCL5*^*+*^), GZMB CD8 EFF (*GZMB*^*+*^*, GZLY*^*+*^), CD8 Ex (*PDCD1*^*+*^*, LAG3*^*+*^), Treg (*FOXP3*^*+*^*, IL2RA*^*+*^), CD4 memory T (*LEF1*^*+*^*, TCF7*^*+*^), CD4 naïve T (*CCR7*^*+*^*, SEEL*^*+*^), NKT (*CD3D*^*+*^*, KLRD1*^*+*^), and NK (*CD3D*^*-*^*, KLRD1*^*+*^), by their indicated canonical mark genes (Fig. 6c, d and Supplementary Fig. 8c). The distributions of all these cell types are observed in each origin tissue and patient, indicating that the data collection was comprehensive and not biased toward any specific cell type or tissue origin (Fig. 6e and Supplementary Fig. 8d). To delineate lineage relationships between CD4^+^ and CD8^+^ T cell subsets, we performed compartment-specific analyses. For CD4^+^ populations, unsupervised clustering revealed a differentiation trajectory from CD4^+^ memory T cells toward Tregs (Fig. 6f, g). In CD8^+^ subsets, trajectory inference identified two divergent paths: (1) a cytotoxic differentiation axis progressing from naïve through memory to terminally differentiated GZMK effector cells, and (2) an exhaustion pathway where GZMK^+^ intermediates transitioned to exhausted CD8^+^ T cells (Fig. 6h, i). Interestingly, all exhausted CD8^+^ cells have a high level of GZMB rather than GZMK (Fig. 6d and Supplementary Fig. 8e). This bifurcation highlights distinct ontogenies for cytotoxic effectors versus exhausted populations.

Among these cells, the MAIT and NKT are mainly distributed in liver metastatic lesions (Fig. 6e). The other subtypes are observed across all tissue origins. The CD8^+^EFF GZMK and CD8^+^EFF GZMB cells are predominant T cell types in these pancreatic tumors (Supplementary Fig. 8d). Interestingly, the expression of GZMK and GZMB genes shows distinct patterns, with a large proportion of cells expressing GZMK but not GZMB, and a smaller proportion expressing GZMB (Fig. 6d, j). To investigate their function, we characterize the representative signatures individually. On the one side, the GSVA analysis shows that inflammatory, cell cycle, glycolysis, and lipid metabolism related signatures are enriched CD8^+^EFF GZMK population (Fig. 6k and Supplementary Fig. 9a). Accordingly, genes related to cytokines (*CXCR4, CXCR6, CCL3L1, CCL4L2, CXCR3*), cell cycle (*DUSP2, DUSP4, DUSP1, FOSB, FOS*) are significantly upregulated in this population (Supplementary Fig. 9b, c). On the other side, signatures related to immunocyte mediated cell toxicity are enriched in CD8^+^EFF GZMB cells (Fig. 6l). In line with this, genes related to cytotoxicity (*FGFBP2, GNLY, GZMB, GZMH, NKG7, PRF1*), T cell activation (*FCGR3A, KLRD1, KLRF1*), and maturation (*KLF2, CD52, ZEB2*) are upregulated in this population (Supplementary Fig. 9c).

To characterize the alteration of these T cell subtypes during PDAC evolution, we calculated the proportions of each population in total immunocytes (CD45^+^ cells). Notably, from HD to non-metastatic primary tumor to metastatic primary tumor to metastatic lesion, a clearly gradual drop of CD8^+^GZMK and CD8^+^Trm is observed (Supplementary Fig. 10a). On the contrary, the variation of other T cell populations is not significant. It indicates that CD8^+^GZMK and CD8^+^Trm cells are susceptible to the lost of DCs. In addition to drops in cell number, we wonder whether or not the remained T cell still keep their bio-functions. The correlation analysis shows that each indicated T cell subtype presents a similar transcriptome signature during the tumor evolution (Supplementary Fig. 10b). The DEG analysis fails to capture functional genes with significant expressional variation (Supplementary Fig. 10c, d). These results suggest that the number, rather than biofunction, of CD8^+^GZMK and CD8^+^Trm cells decreases during tumor evolution.

### Deciphering the heterogeneity of NKs during PDAC malignant transformation

**Fig. 7:**
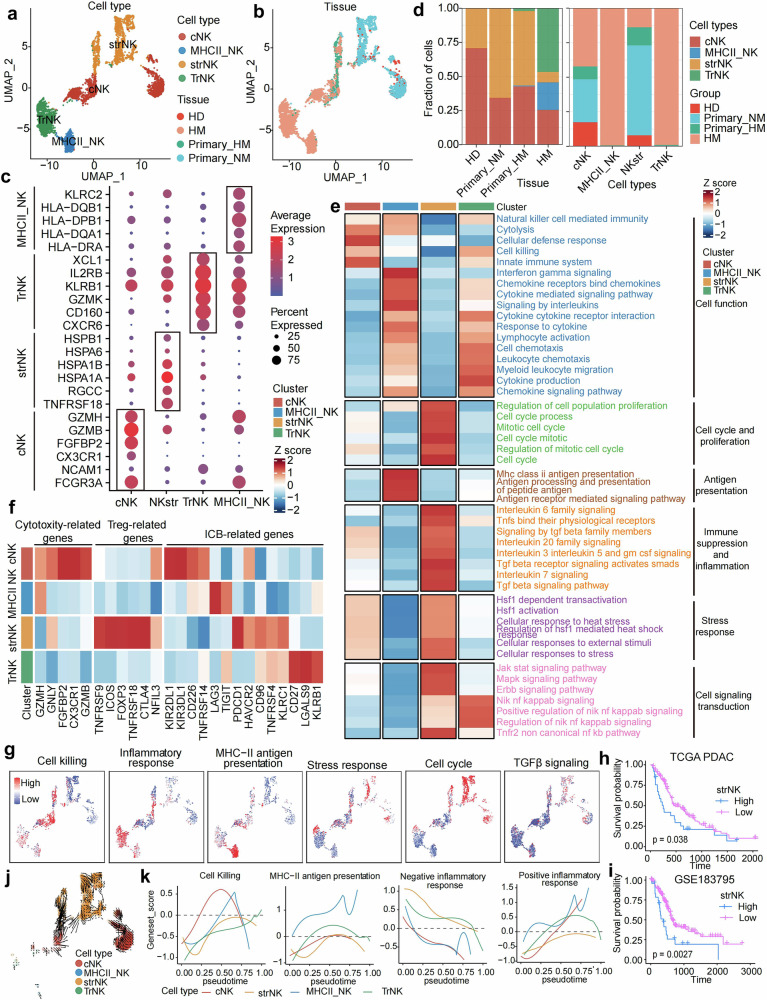
NK cell subtype analysis and functional profiling. **a** UMAP plot presents the sub-clustered NK cells from all biopsies, with cells color-coded according to cell type. **b** The UMAP plot demonstrates the tissue origin of NK cells, showing the distribution across various tissue types. **c** A dot plot exhibits the expression of canonical marker genes corresponding to each NK cell type, with color indicating the average of normalized expression and dot size representing the percentage of marker gene-positive cells. **d** A bar plot displays the proportion of NK cell subtypes within the cohorts HD, Primary_NM, Primary_HM, and HM. **e** A heatmap shows the differentially enriched gene sets between NK cell subtypes, categorized by function: cell function (navy blue), cell cycle (green), antigen presentation (maroon), immune suppression and inflammation (orange), stress response (purple), and cell signaling transduction (magenta). **f** The plot presents the average expression levels of cytotoxicity-related genes, Treg-related genes, and ICB-related genes in each NK cell subtype. **g** The enrichment score of the canonical gene set is projected on the UMAP plot for indicated cell subtypes, color-coded from blue to red to represent the intensity of gene set expression. **h**, **i** Kaplan–Meier plots demonstrate the overall survival of patients with high and low strNK in the TCGA PDAC dataset (upper plot) and GSE183795 (lower plot), with statistical significance assessed using the log-rank test. **j** Velocyto trajectory analysis illustrates the differentiation trajectories of NK cells, with cells color-coded according to their corresponding cell types, revealing the developmental paths. **k** Two-dimensional plots portray expression scores for four representative gene signatures in NK cell subtypes along the inferred pseudotime, providing a temporal view of gene expression dynamics

Based on DEGs and canonical marker genes, total 4,057 NK cells are defined and are divided into 4 categories: conventional NK (cNK, *CX3CR1, FGFBP2, GZMB, GZMH*), stress response NK (strNK, *HSPA1A, HSPA1B, HSPA6, HSPB1*), MHC-II related NK (MHCII NK, *HLA-DRA1, HLA-DQA1,HLA-DPB1, HLA-DQB1, HLA-DRAC*), and tissue-resident NK (trNK, *CXCR6, CD160, GZMK, KLRB1*) (Fig. 7a–c, Supplementary Fig. 11a). The cNK and strNK are observed across HD tissue, primary tumor, and HM lesion. While the MHC-II NK and TrNK exhibit a predominant distribution within HM (Fig. 7d). The cNK cluster shows high expressional levels of cytotoxicity-related genes, such as *CX3CR1, FGFBP2, GZMB, GZMH* (Supplementary Fig. 11b, c). GSVA analysis shows that immune-related cell killing signatures are enriched in cNK, implying their intrinsic tumor immune surveillance function (Fig. 7e). The canonical functional marker genes and enrichment score further confirmed the cellular killer ability of cNK (Fig. 7f, g). On the contrast, trNK shows relatively low cytotoxic function and inflammatory characters. Interestingly, a high level of immune checkpoint gene TIGIT is observed in this population (Fig. 7f), which is in line with previous conclusion drawn from the hepatocellular carcinoma (HCC) atlas investigation.^30^ MHC-II NK, a subset of the trNK cell population, exhibits a significant upregulation of genes associated with antigen presentation, including *HLA-DRA, HLA-DQA1, HLA-DPB1, HLA-DQB1*, and *HLA-DR* (Fig. 7c and Supplementary Fig. 11c). This gene expression profile suggests that MHCII NK may serve a role analogous to that of antigen-presenting cells within the immune system.

Notably, among all NKs, a group of NKs, which show remarkable stress-related heat shock genes and signatures, are identified as strNK. In parallel to the stress response T cell (Tstr),^31^ which was identified as a T cell population related to immunotherapy resistance, strNK shows a discriminate character and function in tumor microenvironment. First, the stress response related genes (*HSPA1A, HSPA1B, HSPA6, HSPB1*), which also serve as maker genes for this cell type, are highly expressed (Fig. 7c and Supplementary Fig. 11c). Second, the proliferation related genes and signatures are highly up-regulated in strNK population (Fig. 7e and Supplementary Fig. 11c). Third, the strNK population show a character of negative immune regulation and immune suppression (Fig. 7e). Interestingly, some canonical Treg related genes,^32^ such as *FOXP3, CYLA4, ICOS*, and *TNFRSF18*, are highly expressed in strNK (Fig. 7f). Moreover, relative low cell killing score and down regulated cytotoxicity related genes indicate that their cytolytic function is deficient (Fig. 7f, g and Supplementary Fig. 11d). Briefly, the strNK is a group of NKs with highly proliferative properties and negative immune regulation but less function of cytolysis. Further clinical survival analysis of PDAC patients with TCGA data set and an independent cohort (GSE183795) indicates that high level of strNK implies a worse prognosis (Fig. 7h, i). In addition, a relative exclusive distribution of immune checkpoints are observed between cNK, trNKs, and strNK (Fig. 7f). *KLRC1* (NKG2A) level is low in the functional population cNK, which partially address the current clinical failure of Monalizumab (monoclonal antibody against NKG2A, NCT02459301, NCT02331875, NCT02557516) in advanced PDAC therapy. On the contrary, the immune checkpoint molecules *KIR2DL1, KIR3DL1*, and *CD226* are relatively high in cNKs (Fig. 7f), suggesting their potential role as therapeutic targets.

The trajectory analysis indicates that cNK, trNKs, and strNK present relative “independent” development road maps, implying their divergent cell fate (Fig. 7j). We further check the representative functions of each population during their life-span developmental process (Fig. 7k). The results showed that the score of cellular killing, antigen presentation, and inflammatory response are highly enriched in cNK, trNKs, and strNK, respectively.

## Discussion

Illuminating the stepwise alterations of balance between immune surveillance and escape during tumor evolution is critical for understanding how the immune system contributes to malignant transition. In this manuscript, we profile the transcriptome of pancreatic tissues collected from healthy donors, PDAC tumors without metastasis, PDAC tumors with liver metastasis, and hepatic metastatic PDAC tumors at single-cell scale. By dissecting immune microenvironment from each representative tumor stage, we unraveled a gradual decline of adaptive immune surveillance along with the malignant development. The mechanism study shows that tumor cell directly triggers apoptosis of DCs through molecular pair ANXA1:FPR1/3. Without antigen cross presentation by DCs, CD8^+^ T cell population drops gradually, which provides an immune exemptional microenvironment.

Principally, tumor escapes immune surveillance through two major approaches: intrinsic and environmental alterations.^33,34^ On the one hand, tumor cells either restrain antigen presentation machinery or engage immune modulatory checkpoints to dampen the cellular immunity-mediated surveillance directly.^33–35^ On the other hand, tumor cells construct an immune suppressive microenvironment by hijacking immune negative regulatory stroma cells such as fibroblast, macrophage, MDSC, etc., through direct cell-cell interaction or paracrine.^33,34^ Due to their pivotal role in adaptive immune surveillance, DCs are suspected to be restrained during tumor cell malignant transition. Previous studies report that tumor cells inhibit DCs maturation and infiltration, as well as suppress immune response function by secretion of cytokines, such as TGF-β or interleukin-10.^36–38^ Tumor cells can also dampen the antigen presentation function of DCs by recruiting immune suppressive cells such as Tregs, MDSCs, and TAMs.^39^ All these regulations on disrupting DCs’ development or inducing their dysfunction are mediated by either cytokines or regulatory cells, which highlight an indirect pattern of interaction between tumor cells and DCs. On the contrary, our study unravels a direct cellular interaction between tumor cells and DCs (ANXA1:FPR1/3), which induces apoptosis of DC. However, whether the decrease of DCs during PDAC evolution is primarily due to ANXA1-FPR1/3-induced apoptosis or tumor microenvironment-mediated inhibition of DC infiltration remains to be further explored.

ANXA1-FPR1/3 is a multifaceted regulator in the tumor microenvironment. Our study reveals a novel mechanism by which tumor cells directly impair DC function through the ANXA1-FPR1/3 interaction. Beyond inducing DC apoptosis, ANXA1-FPR1/3 may play other important roles in immune regulation and inflammation,^40^ such as modulating CD4 T cells differentiation,^41^ promoting M2 macrophage polarization,^42^ as well as inducing neutrophils and DCs apoptosis,^43^ as well as recruitment of DC to the tumor beds.^44^ Thus, the roles of ANXA1-FPR1/3 should be contextualized within the broader landscape of tumor-stroma interactions. For example, galectins, a family of β-galactoside-binding proteins, are well-established mediators of tumor-stroma crosstalk. Galectin-1/3/9, in particular, has been implicated in promoting immune evasion by inducing T cell apoptosis, suppressing DC maturation, and enhancing the immunosuppressive activity of Tregs and MDSCs.^45,46^ The ANXA1/FPR1-3 axis may function in concert with these pathways to create a multifaceted immunosuppressive environment. Future studies should explore potential crosstalk between ANXA1/FPR1-3 and other well-characterized molecular pairs, such as galectins, to better understand their collective contribution to immune evasion in PDAC. Additionally, recent studies have indicated that secreted ANXA1 in plasma may function as a circulating biomarker for the diagnosis of COVID-19 and colorectal cancer,^47,48^ thus suggesting its prospective roles in the diagnosis of PDAC.

We also identified a group of NKs with clear signatures in response to stress, which is negatively correlated with prognosis. In a previous study, Chu et al. reported that CD8^+^ T cells with the stress signatures may potentially mediate the primary resistance to PD-1 blockade therapy, and consequently be correlated with poor response.^31^ Consistently, this strNK population presents a strong character of proliferation, negative immune regulation and reduced cytotoxicity. Additionally, the strNK also highly expressed Treg-related genes, such as *FOXP3, CTLA4, ICOS*, and *TNFRSF18*, which may potentially endow their immune suppression ability. Thus, this dysfunctional NK subpopulation may further contribute to the failure of immune surveillance in PDAC. Characterizing the driver molecules and signaling pathways of this population may provide potential targets for recovering innate immune surveillance.

In conclusion, our study integrates publicly available and in-house datasets to capture PDAC progression. While rigorous batch correction mitigated technical biases, we acknowledge that compositional differences in public datasets (e.g., neutrophil scarcity in CRA001160/GSE205049 due to RNA degradation) may limit resolution for specific cell types. Additionally, the small cohort size may influence calculations of changes in subtype proportions. In the future, larger longitudinal studies are crucial to determine whether our findings are random sampling effects or consistent features of metastatic niche formation. However, key conclusions were validated by in vitro and in vivo studies, ensuring their biological relevance. Our study highlights the dynamic interplay between tumor cells and the immune microenvironment during PDAC progression. The discovery of the ANXA1-FPR1/3 axis as a direct mediator of DC apoptosis offers a novel therapeutic target for disrupting tumor immune evasion. However, the broader implications of this mechanism should be explored in the context of other well-established tumor-stroma interactions. Larger longitudinal cohorts also are essential to statistically resolve whether these represent stochastic sampling effects or reproducible hallmarks of metastatic niche formation. Furthermore, the identification of dysfunctional NK cell populations underscores the need for strategies to rejuvenate both adaptive and innate immune responses in PDAC. Together, these findings provide new insights into the complex immune landscape of PDAC and pave the way for the development of more effective immunotherapies.

## Materials and methods

### Patients and specimens

This study was approved by the Ethics Committee on Biomedical Research of West China Hospital and we complied with all relevant ethical regulations (ethical approval number: No. 2021[484]). The patients and healthy donors described in this study provided written informed consent. We collected 6 PDAC liver metastatic patient-matched primary and metastatic lesions, 2 patients without metastasis, and 2 healthy pancreatic tissues without tumor to perform single-cell RNA-seq and exome sequencing.

### Cell lines and culture conditions

The mouse PDAC cell line KPC was obtained from the Department of Pancreatic Surgery at West China Hospital (Chengdu, China). HEK-293T and DC2.1 cell line were purchased from the National Collection of Authenticated Cell Cultures (Shanghai, China). Mouse KPC and DC2.4 cells were maintained in RPMI-1640 medium (Gibco) with 10% FBS. HEK-293T cells were maintained in DMEM medium (Gibco) with 10% FBS. Mouse BMDC cells were isolated from bone marrow of C56BL/6 mice and cultured in RPMI-1640 medium with mGMCSF (20 ng/ml) and mIL-4 (10 ng/ml). All cells were cultured aseptically at 37 °C in humidified incubators with 5% CO_2_.

### Animals and housing conditions

Six weeks-old female C57BL/6 mice (specific pathogen-free conditions, SPF) were purchased from Beijing Vital River Laboratory Animal Technology Co., Ltd (Beijing, China). The animals were housed and maintained under SPF conditions in facilities and treated humanely throughout the studies. All animal experiments were performed according to the protocols approved by the Ethics Review Committee of Animal Experimentation of Sichuan University (ethical approval number: No.20240301156). All our animal-handling procedures were performed according to the Guide for the Care and Use of Laboratory Animals of the National Institutes of Health and the guidelines of the Animal Welfare Act.

### Preparation of single-cell suspensions

We collected NM, HM, and LM samples from PDAC patients through laparoscopic surgery. The HD samples were collected from non-malignant pancreatic tumor patients who received pancreatoduodenectomy or distal pancreatectomy. The methods of single cell dissociation are similar with our previous study.^22–24^ Briefly, cut the tissues with scissors on ice and transfer them to a 50 ml centrifuge tube with 20 ml digestive enzyme containing (Gibco) 0.25% trypsin, 0.4 mg/ml collagenase type I, and type IV (Gibco). Then the tissues were incubated for 15 min at 37 °C, with manual shaking. After dissociation, single-cell suspensions were neutralized with ice-cold DMEM (containing 10% fetal bovine serum) and filtered with a 70-μm cell strainer to remove large pieces of debris. Next, the single-cell suspension was centrifugated at 500 × *g* for 5 min and resuspended in red blood cell lysis buffer to remove red blood cells. Following a 5 min incubation on ice, single-cell suspensions were washed twice with HBSS and resuspended in HBSS (containing 0.1%BSA) buffer. The resuspended cells were then sorted on flow cytometer (BD Biosciences, SanJose, CA) to isolate high bioactivity indicated cells. After sorting, cells were counted and assessed for viability (> 90%) with Trypan blue using a Countess II automated counter. The cells were then resuspended in HBSS for single-cell RNA-seq.

### Preparation of scRNA-seq libraries

Single-cell RNA-seq libraries were generated by the 10× Genomics Chromium 3′ Gene Expression Kit V3. Briefly, cell suspensions were loaded onto a Chromium Single-cell Chips with a capture target of 6000 ~ 8000 cells per sample. Libraries were constructed according to the provided protocol and sequenced on Illumina HiSeq 4000 platform with a targeted sequencing depth of 100,000 reads per cell. Then, the fastq files obtained following demultiplexing with Illumina Bcl2fastq were used as inputs to downstream analysis.

### Data collection

In this study, we conducted a comprehensive analysis of the TME of PDAC using single-cell RNA sequencing (scRNA-seq) data from 51 samples across 34 individuals. The dataset comprised four distinct sources: a newly generated dataset that consisting of healthy donor pancreatic (HD) tissues and non-metastatic pancreatic primary tumors (primary_NM), our previously generated dataset (OMIX: OMIX002487) that included primary tumor with hepatic metastasis (primary_HM), and matched hepatic metastases (HM) lesions, and two public datasets (CRA001160^20^ and GSE205049^12^) that also contained samples from HD tissue and primary_NM tumor. Both the newly added dataset and OMIX002487 dataset were generated in parallel using the same 10× Genomics Chromium 3′ v3 workflow and Illumina HiSeq 4000 sequencing, ensuring technical equivalence and minimal batch effects. The 51 samples consisted of 22 HD tissues, 17 primary_NM tumors, 6 primary_HM tumors, and 6 HM tumors. All the data in the four datasets were constructed using the 10× Genomics technology. The CRA001160 dataset predominantly captured non-immune cells, while the GSE205049 dataset consisted exclusively of immune cells. In our dataset (OMIX: OMIX002487), samples P1, P2, P3, P4, and P6 contained both immune and non-immune cells, whereas sample P5 mainly comprised non-immune cells, as CD45- cells were sorted for single-cell RNA sequencing.^22^ Human bulk RNA sequence data for survival analysis was downloaded from GEO database (GSE183795)^49^ and The Cancer Genome Atlas Program (TCGA) database.

### Single-cell RNA sequencing data processing

The single-cell RNA sequencing (scRNA-seq) data of paired pancreatic ductal adenocarcinoma (PDAC) tumor samples were processed using the cellranger software suite, version 5.0.0, provided by 10× Genomics. This software is specifically designed for the analysis of single-cell transcriptomic data generated through the Chromium platform, ensuring compatibility and precision in data handling. The initial step in our analysis involved the alignment of raw sequencing reads to the GRCh38 human genome reference using cellranger’s alignment module. This module is optimized for accurate mapping of short reads to the reference genome, allowing for the identification of transcriptomic features at the single-cell level. Following alignment, the cellranger count pipeline was utilized to quantify gene expression across the samples. This pipeline generates feature-barcode matrices, which are crucial for subsequent analytical steps. These matrices provide a comprehensive overview of the gene expression landscape within each cell, facilitating the identification of cellular heterogeneity and transcriptomic variability.

### Quality control measures

To ensure the reliability and integrity of our scRNA-seq data, a rigorous quality control protocol was implemented. Cells with an abnormal number of expressed genes, indicative of low-quality cells or potential doublets, were filtered out. Specifically, cells expressing more than 7500 genes or fewer than 200 genes were excluded from further analysis. Additionally, cells with mitochondrial gene expression levels exceeding 25% were removed from the dataset. Elevated mitochondrial gene expression is often associated with cell stress or damage, which could skew the interpretation of the data. To further refine the dataset, genes that were expressed in fewer than three cells within a sample were also eliminated. The identification of doublets, which are two cells captured in the same droplet, was performed using a doublet detection algorithm that identifies cells expressing multiple cell markers. The presence of doublets can confound downstream analyses, particularly in clustering and cell type identification. After applying these quality control measures, a total of 122, 479 single cells from 49 samples were retained for analysis.

### Batch correction

The gene expression matrices were imported into the Seurat package (version 4.0.0)^50^ for preprocessing. We employed the robust principal component analysis (RPCA) method as implemented in Seurat. RPCA is a sophisticated technique that decomposes the gene expression data into low-rank and sparse components. The low-rank component captures the majority of the biological signal, while the sparse component accounts for the batch effects and other technical artifacts. By applying RPCA, we effectively separated the biological variation from technical noise, allowing for a more accurate representation of the underlying cellular heterogeneity. The success of batch effect removal was evidenced by the overlap of our self-measured data with public datasets, specifically the HD and Primary_NM samples. This overlap indicated that our batch correction strategy was effective in harmonizing the datasets and reducing potential confounding factors.

To rigorously validate the robustness of batch effect correction, we implemented two complementary integration methods - Canonical Correlation Analysis (CCA) and Harmony using default parameters. Integration efficacy was assessed through the low-dimensional UMAP visualization. Seamless mixing of immune cells across datasets with no residual technical clustering.

### Dimensionality reduction and clustering

Following stringent quality control procedures on cells and genes, we advanced to dimensionality reduction and unsupervised clustering using Seurat (version 4.0.0) package.^50^ We initiated the analysis with global-scaling normalization through the “LogNormalize” method, which standardizes gene expression across cells relative to total expression, scales by a factor of 10,000, and applies a logarithmic transformation. Subsequently, the FindVariableFeatures function pinpointed 2000 highly variable genes, which were instrumental for capturing the biological variance within the dataset. These genes guided the subsequent scaling to z-scores via the ScaleData function, priming the data for principal component analysis (PCA) with the RunPCA function, focusing on the first 50 principal components as delineated by an Elbow plot. Employing a graph-based clustering strategy, we utilized the FindNeighbors function to construct a KNN graph, refining edge weights to delineate cell-cell relationships based on the top 50 principal components. The Louvain algorithm was then engaged through the FindClusters function, set at a resolution of 1, to iteratively cluster cells. Finally, UMAP was applied to visualize the low-dimensional representation of the scRNA-seq data, leveraging the 50 principal components to offer a comprehensive view of cellular heterogeneity, thereby facilitating the identification of distinct cellular populations and underlying biological processes.

### Cell type annotation

After quality control and unsupervised clustering, cell annotation was conducted using the sciBet (version 1.0) R package,^51^ which integrates conical marker genes and inferCNV results for cell type identification. Initially, cells were broadly classified into epithelial cells (positive for EPCAM, KRT8, and KRT18), fibroblasts (positive for FAP and COL1A1), endothelial cells (positive for VWF and PECAM1), and immunocytes (positive for PTPRC). Subsequently, the immune cell subset was subjected to re-clustering and further annotation, leveraging the expression of canonical marker genes and knowledge of tissue origin to delineate sub-cell types.^22^

### Gene set enrichment analysis

For the enrichment analysis of our scRNA-seq data, we employed gene sets from HALLMARK, C2.CP.KEGG, C2.CP.REACTOME, and C5.BP, sourced from the Molecular Signatures Database (MSigDB, version 7.1).^52^ Utilizing the SingleCellSignatureExplorer (version 3.6),^53^ we computed single-cell signature scores to quantify the activity of these gene sets within each cell. Subsequently, the limma (version 3.42.0) R package^54^ was applied to conduct differential expression analysis on the computed signature scores, identifying gene sets with adjusted P values (p.adj) less than 0.05 as significantly differential.

### Infer the copy number variation from sc-RNAseq data

Copy Number Variations (CNVs) were inferred from the scRNA-seq data using CopyKAT (v.1.0.5) R package,^55^ a computational tool designed for CNV detection in single-cell data. CopyKAT was run with the default parameters. The software utilizes a Bayesian hierarchical model to estimate the copy number states and generates a final CNV call set. To calculate the Copy Number Variation (CNV) score for each cell, we applied a function that evaluates the proportion of elements in each cell that deviate from the equilibrium state by more than 0.05. The CNV score for cell *j* is computed using the formula:

$${CNV\,score}_{j}=\frac{{\sum }_{i=1}^{n}1(|{x}_{ij}|\, > \,0.05)}{n}$$

where *xij* represents the *i*-th element of cell *j CNVs*, and *n* is the total number of elements in cell *j*. This score reflects the relative frequency of aberrant values exceeding the threshold in each column.

### Developmental trajectory analysis

To delineate cellular developmental trajectories in our scRNA-seq dataset, we implemented a dual analytical framework leveraging RNA velocity and pseudotemporal ordering. Primary trajectory analysis was performed using Velocyto (v0.17) with default parameters, which calculates transcriptional dynamics through spliced/unspliced mRNA ratios to predict future cell states. Given the ontogenetic heterogeneity between pancreatic-derived (Primary_NM/HM) and hepatic-infiltrating immune populations, we restricted developmental trajectory analysis to HD, Primary_NM, and Primary_HM samples where cellular provenance could be unambiguously attributed to pancreatic tumor evolution. These velocity vectors were visualized via scVelo’s (v0.2.4) streamplots embedded in UMAP projections, where arrow directionality and length quantitatively represent differentiation probabilities. To validate trajectory robustness, we employed Monocle 3 (v0.2.3) as an orthogonal validation tool, constructing cell fate maps through reversed graph embedding of highly variable genes. The convergence of pseudotime root-leaf ordering in Monocle 3 with Velocyto’s velocity streamlines confirmed trajectory reliability.

### Cell-cell interaction analysis

To investigate the ligand-receptor interactions between tumor cells and immunocytes from our single-cell RNA-seq data, we utilized CellPhoneDB (version 2.0),^56^ a bioinformatics tool that facilitates the analysis of cell-cell communication. The tool’s reference repository, curated from databases such as UniProt, Ensembl, PDB, the IMEx consortium, and IUPHAR, encompasses 1396 receptor-ligand interactions. By querying our scRNA-seq data against this repository, we identified potential interactive molecules. These were then characterized based on two criteria: 1) the mean expression value of ligand-receptor pairs within each cell type, reflecting the strength of interaction, and 2) the *p*-value derived from an empirical shuffling algorithm, indicating the statistical significance of the interaction. We focused on cell-cell contact ligand-receptor interactions as annotated by the CellChat database. Interaction pairs were considered significant if they met the threshold of *p* < 0.05 and a mean expression value greater than 0.5. The interaction weight score for each cell-cell interaction was calculated as the sum of gene expression counts for all significant ligand-receptor pairs, providing a quantitative measure of the interaction strength between different cell types.

### Survival analysis

To study the effect of strNK on survival of cancer patients, we conducted survival analysis of public data in TCGA and GEO. We commenced by identifying the top 100 highly expressed genes, or marker genes, for each cell subtype from the scRNA data. These gene expression profiles were then utilized as the reference input for CIBERSORTx, a computational tool designed to infer cell type abundances from bulk gene expression data. Subsequently, we processed the gene expression matrices from TCGA PDAC and GSE183795 datasets as ‘Matrix’ inputs for the CIBERSORTx cell fraction analysis. After leveraging the strNK cell proportion derived from the results, we employed the surv_cutpoint function from the survminer R package to stratify the samples into distinct groups based on strNK cell prevalence. The stratification outcomes were subsequently visualized using the ggsurvplot function, which facilitated the generation of Kaplan–Meier survival curves.

### Cell transfections

Mouse ANXA1 genes were synthesized and subcloned into lentivirus vector pRRLsin.cPPT.CMV.Blasticidin (denoted as CPPT). shRNA (knockdown) sequences specifically targeted to ANXA1 were synthesized and subcloned into PLKO vector to generate lentiviruses. Recombinant lentiviruses were generated by third-generation lentiviral packaging using human embryonic kidney (HEK) 293T cells. HEK293T cells were transfected using calcium phosphate. Then the lentiviruses were harvested and transfected with tumor cells. After 24 h, the tumor cells were screened with blasticidin (for CPPT vector, Invivogen, #ant-bl-1) or puromycin (for PLKO vector, Selleck, #S7417) to obtain highly transduced cells.

### Flow cytometry analysis the apoptosis of DCs

The activated BMDCs were seeded into 12-well plates and cultured overnight. The cells were then treated with indicated doses (0, 5, 10, and 20 μg/ml) of ANXA1 protein (MedChemExpress, HY-P72078) for 24 h, based on previous studies demonstrating the effective modulation of DC function within this concentration range.^41,43,57^ After incubation, cell apoptosis was detected with Annexin V-FITC/PI Apoptosis Detection Kit according to the manufacturer’s instructions (Beijing 4A Biotech Co., Ltd., #FXP018).

### Western blot analysis

DC2.4 and BMDC cells were incubated with ANXA1 protein (10 μg/ml) for 24 h. Then, the cells were harvested and lysed with RIPA buffer in the presence of protease and phosphatase inhibitor at indicated time point. Protein lysates were electrophoresed and transferred to PVDF membranes (Bio-Rad). Western blots were performed using the following antibodies: cle-caspase3 (Cell Signaling Technology, #9664), BCL-2 (Abclonal, #A19693), MCL-1 (Abcam, #ab32087), and TUBULIN (Beijing Zhong Shan -Golden Bridge Biological Technology, #TA-10).

### Animal treatment study

For the subcutaneous flank tumor model, KPC cells (5 × 10^5^ cells) with or without ANXA1 knockdown were implanted subcutaneously in the right flank of C57BL/6 mice. When the tumor volume reached ~80 mm^3^, animals were randomized into different treatment groups and intravenously injected with anti-PD-1 blockade antibody (2.5 mg/kg). A total of three doses of anti-PD1 antibody were administrated every three days. The tumors were measured every three days using a Vernier caliper, and tumor volumes were calculated using the formula: Volume = (tumor length) × (tumor width)^2^/2. At the end of treatment, the tumor tissues were collected for IF and flow cytometry analysis.

To mimic the human pancreatic cancer microenvironment, we also established an orthotopic PDAC mouse model by injecting luciferase-tagged KPC cells (KPC-Luc, 1.5 × 10^5^ cells) with ANXA1 overexpression, ANXA1 knockdown, or empty vector into the pancreas tissues of mice. Anti-PD-1 treatment (2.5 mg/kg, i.v.) was administered every 6 days after 5 days of the tumor inoculation. After twenty days, the mice were intraperitoneal injected with D-luciferin potassium (150 mg/kg) and analyzed by IVIS Spectrum Imaging System. After imaging, mice were sacrificed, and the tumor tissues were harvested for pathological examination and flow cytometry analysis.

### Immunofluorescence (IF) and multiplex immunohistochemistry (mIHC) staining

To prepare samples for IF and mIHC staining, tissue samples were fixed in 10% formalin for 48 h, followed by embedding in paraffin. Tissue sections of 4-μm thickness were mounted on glass slides for IF and mIHC staining according to standardized protocols. First, the slides were deparaffinized and incubated in 3% hydrogen peroxide. Antigen retrieval was performed using either EDTA buffer (pH 9.0) or citric acid (pH 6.0). The sections were preincubated with an antibody diluent to block nonspecific antibody binding sites and then incubated sequentially with the primary and secondary antibodies according to the experimental protocol for IF. For mIHC staining, an Opal Polaris™ 7-Color Manual IHC kit was employed following the manufacturer’s instructions. After incubating with the primary antibody, the slides were treated with the secondary antibody and underwent tyrosine signal amplification. Subsequently, the sections were subjected to microwave heat repair, and a second round of staining was performed. Once all indicators were stained, 4′,6-diamidino-2-phenylindole (DAPI) was used to label the nuclei. Multispectral slice scanning and image analysis were conducted using Vectra Polaris (PerkinElmer) and Qupath (version 0.3.0). IF and mIHC staining utilized the following antibodies: cleaved caspase-3 (Cell Signaling Technology, #9664, 1:200), TNF-α (Abcam, #ab1793, 1:200), CD11c (Cell Signaling Technology, #45581, 1:400), ANXA1 (Cell Signaling Technology, #32934, 1:400), and CK8/18 (Abcam, #ab17139, 1:100).

### Flow cytometry analysis

Tumor tissues from subcutaneous and orthotopic pancreatic ductal adenocarcinoma (PDAC) were subjected to flow cytometry analysis. Tumor tissues were dissected into small pieces and digested in RPMI medium containing 0.5 mg/mL collagenase type I and type IV (Gibco) at 37 °C for 20 min. Digested tissues were passed through a 70 μm cell strainer, and single-cell suspensions were washed twice with D’-HANKS buffer. To exclude dead cells, the cells were stained with the Zombie UV™ Fixable Viability Kit (BioLegend, #423108) and incubated with Fc-Block (TruStain fcX™ anti-mouse CD16/32, clone 93, BioLegend) for 15 min to prevent nonspecific binding. Subsequently, fluorophore-conjugated antibodies (BioLegend) were used for a 30-min staining on ice. The antibodies included CD45-APC-Cy7 (#103116), CD3-PerCP-Cy5.5 (#100275), CD8-Spark UV™ 387 (#100798), CD4-Alexa Fluor® 700 (#116021), CD11c-BV510 (#117338), and CD80-PE/Dazzle™ 594 (#104738). Multi-parameter staining identified the following populations: (1) tumor-infiltrating CD3^+^ T cells (CD45^+^CD3^+^), (2) tumor-infiltrating CD8^+^ T cells (CD45^+^CD3^+^CD8^+^), (3) tumor-infiltrating CD4^+^ T cells (CD45^+^CD3^+^CD4^+^), (4) tumor-infiltrating DCs (CD45^+^CD11c^+^), (5) mature DCs (CD11c^+^CD80^+^). The cells were analyzed using a BD FAcsymphonyA5 SE flow cytometer, and the data were processed with FlowJo v10 software.

### Statistical analysis

For the analysis of our single-cell RNA-seq data, we employed the R software environment (version 4.0), leveraging a suite of specialized packages to conduct our statistical evaluations. Kaplan–Meier survival curves, a standard tool for illustrating survival probabilities over time, were generated using the survival and survminer R packages. To handle flow cytometry data, we utilized FlowJo_V10, a dedicated software for complex cytometric data analysis. For the differential gene expression analysis, we applied the Wilcoxon rank-sum test to assess the significance of gene expression differences, while the two-sided Wilcoxon test was employed for testing the significance of individual gene expression levels. When comparing two groups, we used the independent-samples t-test. We established stringent statistical significance thresholds at *p < 0.05, **p < 0.01, *** *p* < 0.001, and **** *p* < 0.0001 to ensure robust findings.

## Supplementary information

Supplementary Figures

## Data Availability

The raw sequencing data that support the findings of this study are deposited at the National Genomics Data Center (NGDC, https://ngdc.cncb.ac.cn/) under project: PRJCA013942 (https://ngdc.cncb.ac.cn). The processed data of scRNA have been deposited at NGDC under project: PRJCA013605. The gene expression profiles and clinical survival data of PDAC in the TCGA dataset were obtained from the UCSC Xena platform (https://xenabrowser.net/hub/), a publicly accessible genomic database. All data supporting this study are available on the NGDC website or provided in the Supplementary Materials.
