# Supplementary material for: Single-cell transcriptional dissection illuminates an evolution of immunosuppressive microenvironment during pancreatic ductal adenocarcinoma metastasis
Source: Signal Transduct Target Ther. 2025 Jun 9;10:182. doi: 10.1038/s41392-025-02265-0 (PMC12146409; doi:10.1038/s41392-025-02265-0)
Supplement: Supplementary file 1 — Supplementary Figures [file 41392_2025_2265_MOESM1_ESM.docx]

Supplementary Materials for

Single-cell transcriptional dissection illuminates an evolution of immunosuppressive microenvironment during pancreatic ductal adenocarcinoma metastasis

Xiaowei Liu, Jinen Song, Meiling Yuan, Huihui Li, Leyi Tang, Fengli Zuo, Xinmin Wang, Xueyan Wang, Qian Xiao, Li Li, Xinyu Liu, Zhankun Yang, Jianlin Wu, Jing Jing, *, Xuelei Ma, * and Hubing Shi

Correspondence to: shihb@scu.edu.cn (H.S.), drmaxuelei@gmail.com (X.M.), jingjing@wchscu.edu.cn (J.J.)

**This PDF file includes:**

Figures. S1 to S11


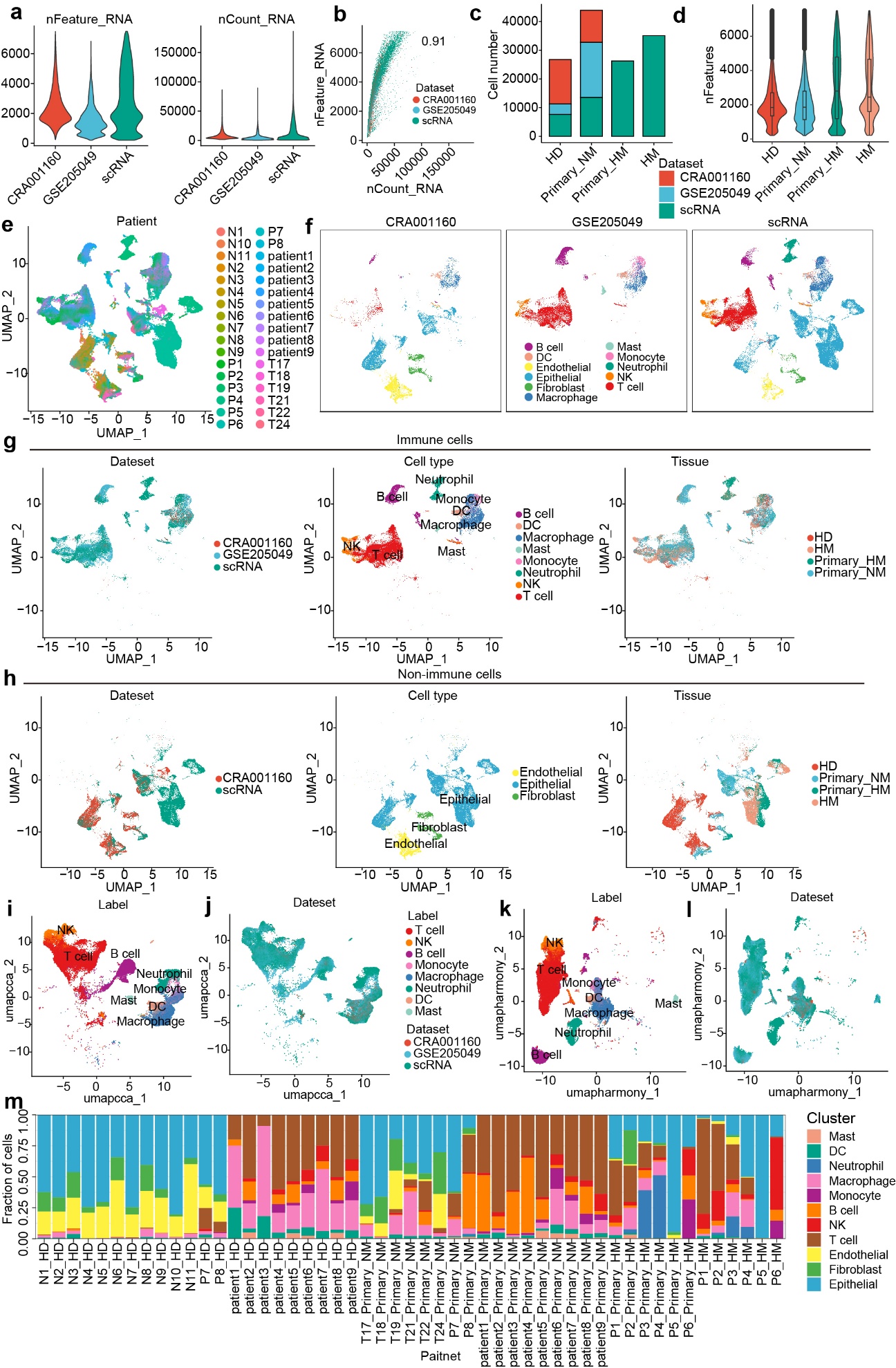


**Supplementary Figure 1. Quality control and cell type distribution**

(a) Violin plots illustrate the quality control metrics for the number of features (nFeature) and number of counts (nCount) per cell, showing the distribution and density of gene expression measurements.

(b) FeatureScatter plot depicts the relationships between nCount and nFeature of RNA for each tissue group, providing a visual assessment of data dispersion and outliers.

(c) A bar plot presents the count of cells measured across the four tissues, offering a quantitative overview of the sample sizes.

(d) A violin plot showing the distribution of feature counts (nFeature_RNA) per cell across the four tissue types in the combined dataset.

(e) The UMAP plot displays all sequenced cells differentiated by patient origin.

(f) UMAP plots display all sequenced cells differentiated by dataset origin. Colors present cell types.

(g) The UMAP plots present the immune cell subtypes distribution after RPCA batch correction.

(h) The UMAP plots present the non-immune cell subtypes distribution after RPCA batch correction.

(i, j) CCA-based integration results of immune subtype preservation (i) and cross-dataset mixing efficiency (j).

(k, l) Harmony-processed embeddings demonstrating lineage-resolved immune clustering (k) and batch-agnostic dataset alignment (l).

(m) A bar plot quantifies the proportion of each cell subtype for each patient, revealing the distribution of cellular populations within individual cases.

**
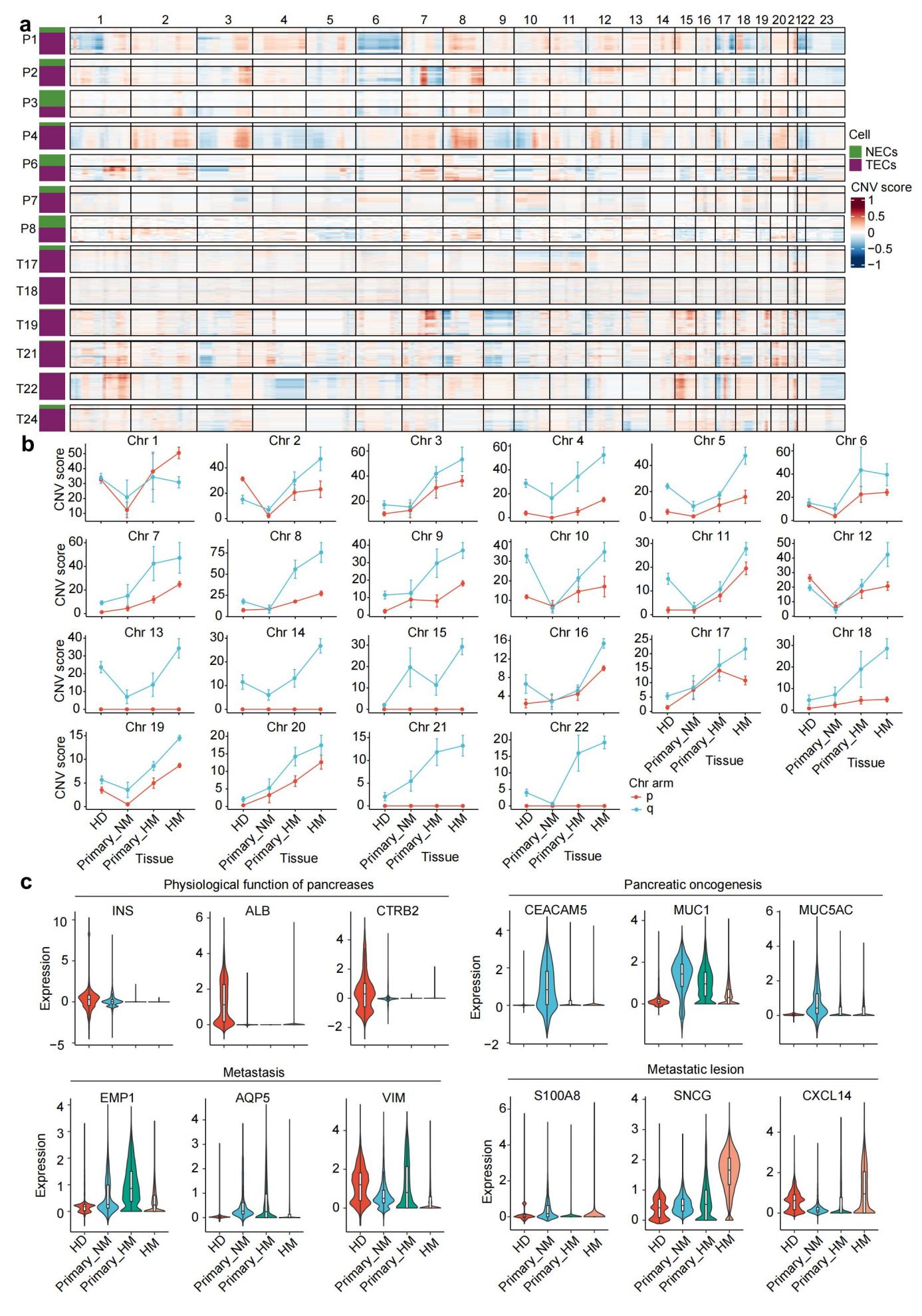
**

**Supplementary Figure 2. Distinguish between malignant and normal epithelial cells**

(a) The heatmap displays the CopyKAT results for epithelial cells from tumor samples, with malignant tumor cells indicated in purple. The chromosome scale is annotated at the top for chromosomal positioning.

(b) Chromosome line plots depict the chromosomal distribution of CNV scores in epithelial cells across four sample groups: HD, Primary_NM, Primary_HM, and HM. The color corresponds to the p arm and q arm of the chromosome.

(c) Bar plots show the expression levels of marker genes characteristic of TECs and HD NECs.


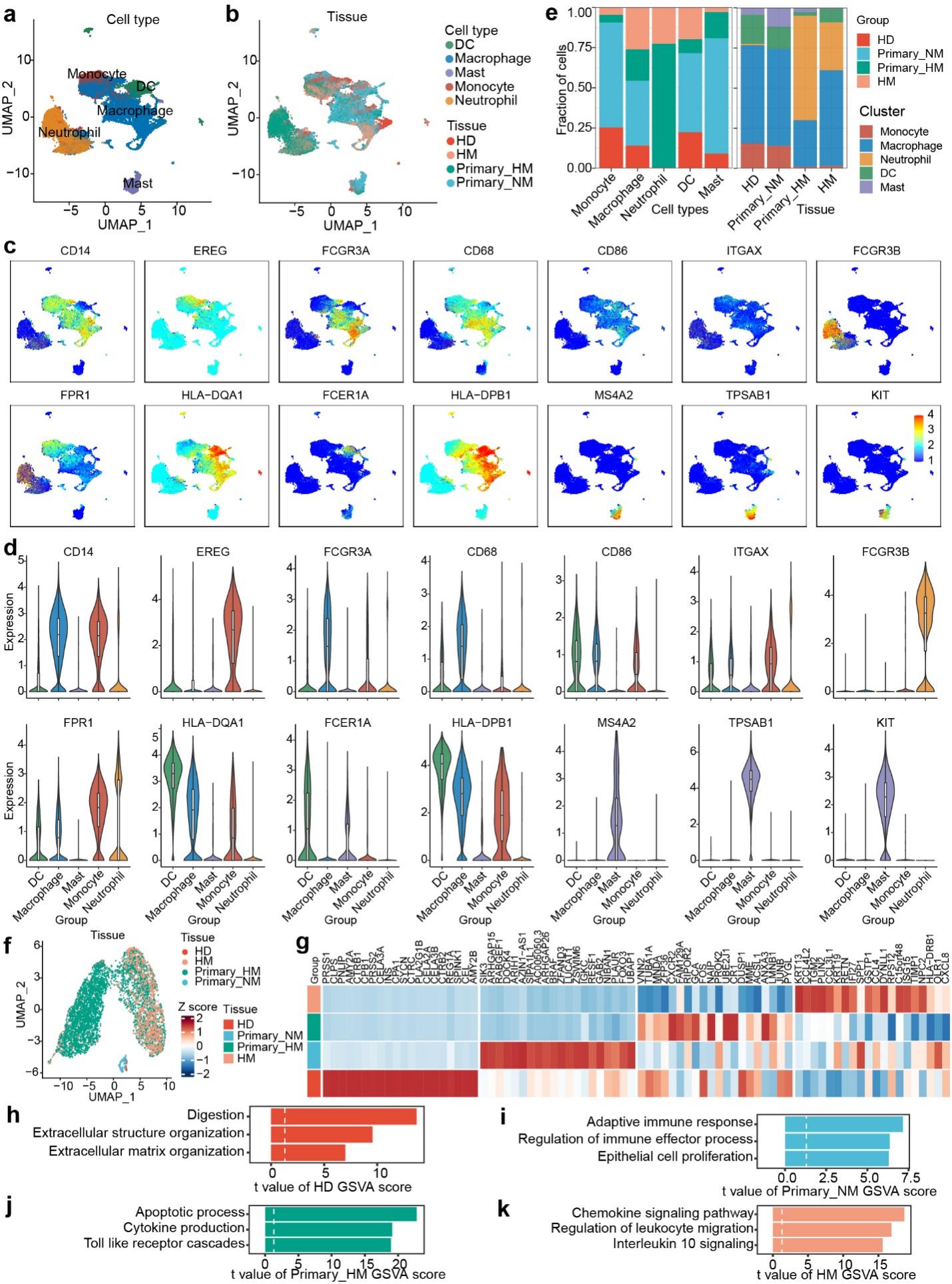


**Supplementary Figure 3. Analysis of myeloid cell subtype**

(a) The UMAP projection displays the sub-clustered myeloid cells from all biopsies, with clusters differentiated by color according to cell type.

(b) The UMAP plot illustrates the tissue origin of all myeloid cells, providing a visual representation of their distribution across tissue types.

(c) The UMAP plots project the expression of marker genes for the indicated cell subtypes, color-coded from blue to red, with five cell types identified: macrophage, DC, neutrophil, monocyte, and mast cells.

(d) Violin plots depict the expression levels of marker genes characteristic of each myeloid cell subtype.

(e) A bar plot quantifies the proportion of each cell type within the cohorts HD, Primary_NM, Primary_HM, and HM, offering insights into their relative abundance across different conditions.

(f) UMAP projection illustrating neutrophil distribution across all biopsy samples, color-coded by tissue origin.

(g) Heatmap of the top 20 differentially expressed genes in neutrophils from HD, Primary_NM, Primary_HM, and HM groups, with color intensity indicating z-scores of gene expression.

(h-k) Pathway activity profiles of neutrophils in HD (h), Primary_NM (i), Primary_HM (j), and HM (k) groups, analyzed using gene set enrichment analysis.


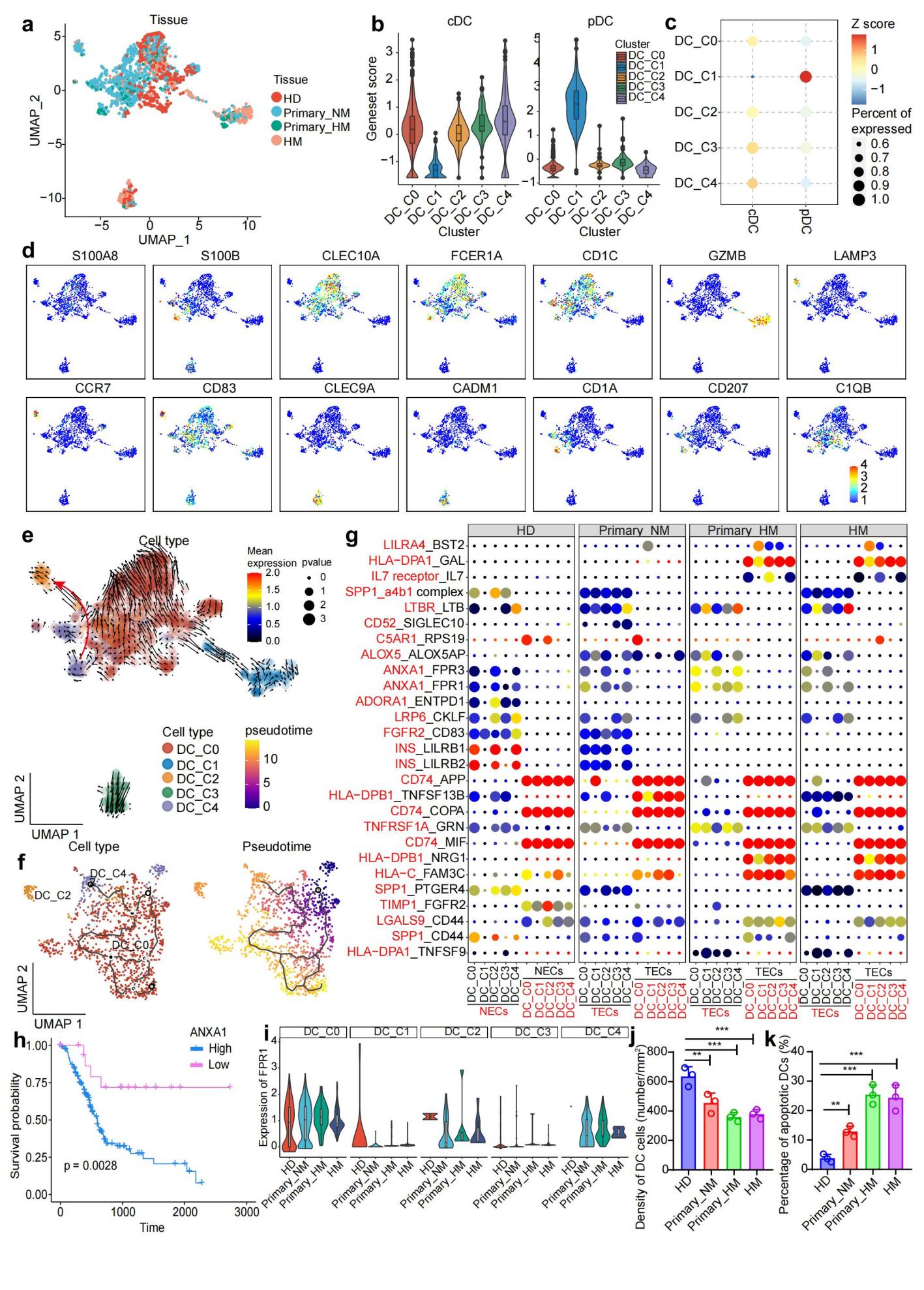


**Supplementary Figure 4. DC subtype analysis and interaction profiling**

(a) A UMAP plot presents the sub-clustered DCs from all biopsies, with cells color-coded according to tissue origin for a clear distinction between tissue types.

(b) Violin plots illustrate the geneset scores for each DC subtype, providing a measure of the expression levels of specific biological pathways within each subtype.

(c) A bubble heatmap represents the scores of canonical signatures corresponding to each DC cell type, where color reflects the z-score of the geneset score, and dot size indicates the percentage of signature-positive cells.

(d) The UMAP plot projects the expression of marker genes for the indicated DC subtypes, color-coded from blue to red.

(e, f) Velocyto (e) and·Monocle3 (f) algorithms calculate·the·evolution of DCs from HD, Primary_NM, and Primary_HM tissues.

(g) An overview of ligand-receptor pairs between TECs / HD NECs and DC subtypes across HD, Primary_NM, Primary_HM, and HM lesion is presented. Color intensity signifies the mean expression of ligand and receptor genes, while dot size corresponds to the statistical significance of the interactive molecular pairs.

(h) Kaplan-Meier survival analysis comparing overall survival between patients with high and low ANXA1 expression in the TCGA PDAC cohort, with statistical significance evaluated by log-rank test.

(i) A violin plot displays the expression levels of FPR1 on DC subtype across different tissues.

(j, k) The densities of DCs (CD11c^+^, j) and the percentage of apoptotic DCs (cleaved caspase-3^+^ CD11c^+^ cells, k) in each tissue of Figure 4h were quantified.

**
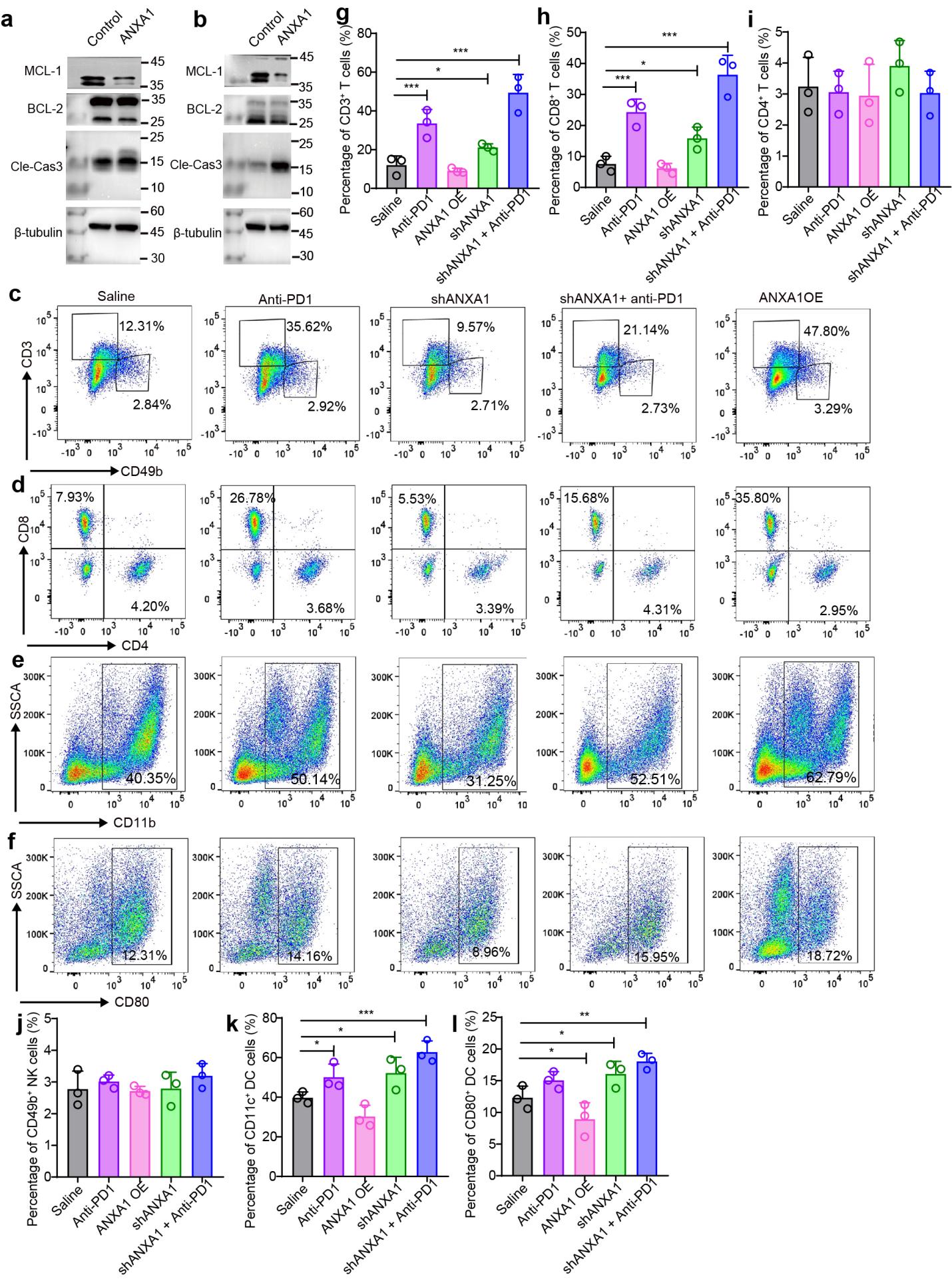
**

**S****upplementary Figure 5. Analyzing the function of ANXA1 in vitro and mouse PDAC subcutaneous tumor model.**

(a, b) The original western blot membranes were used for Figures 5c and 5d.

(c-f) Flow cytometry analysis was conducted on tumor-infiltrating CD3^+^ T cells (c), NKs (CD3^-^CD49b^+^, c) CD8^+^ cytotoxic T cells (CTLs, CD3^+^CD8^+^, d), T helper cells (CD3^+^CD4^+^, d), total DCs (CD45^+^CD11c^+^, e), mature DCs (CD11c^+^CD80^+^, f) in subcutaneous PDAC tumors, with representative scatter plots displayed.

(g-l) The percentages of CD3^+^ T cells (g), CD8^+^ cytotoxic T cells (h), T helper cells (i), total DCs (j), mature DCs (k), and NKs (l) within the total CD45^+^ immune cell population were quantified. The quantitative results were presented as mean ± SD, *P<0.05, **P<0.01, and ***P<0.001.


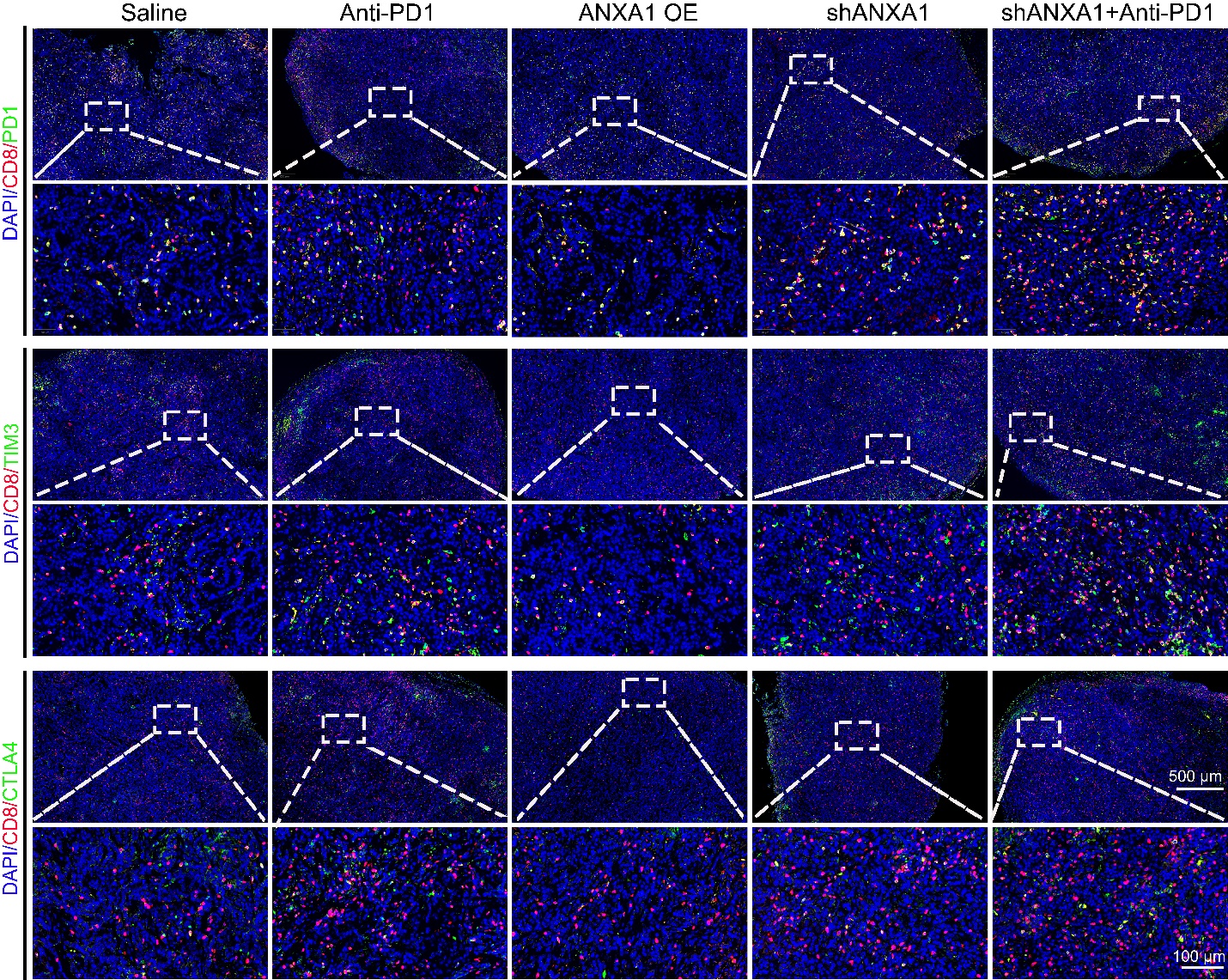


**Supplementary Figure 6.** **Immunofluorescence staining of CD8^+^ T cells co-expressing exhaustion markers.** Nuclei were stained with DAPI (blue), CD8^+^ T cells were labeled with an anti-CD8 antibody (red), and exhaustion markers were detected using anti-PD1, anti-TIM3, and anti-CTLA4 antibodies (green). Triple-positive cells (blue/red/green) indicate CD8^+^ T cells exhibiting an exhausted phenotype. Scale bar, 500 μm or 100 μm.


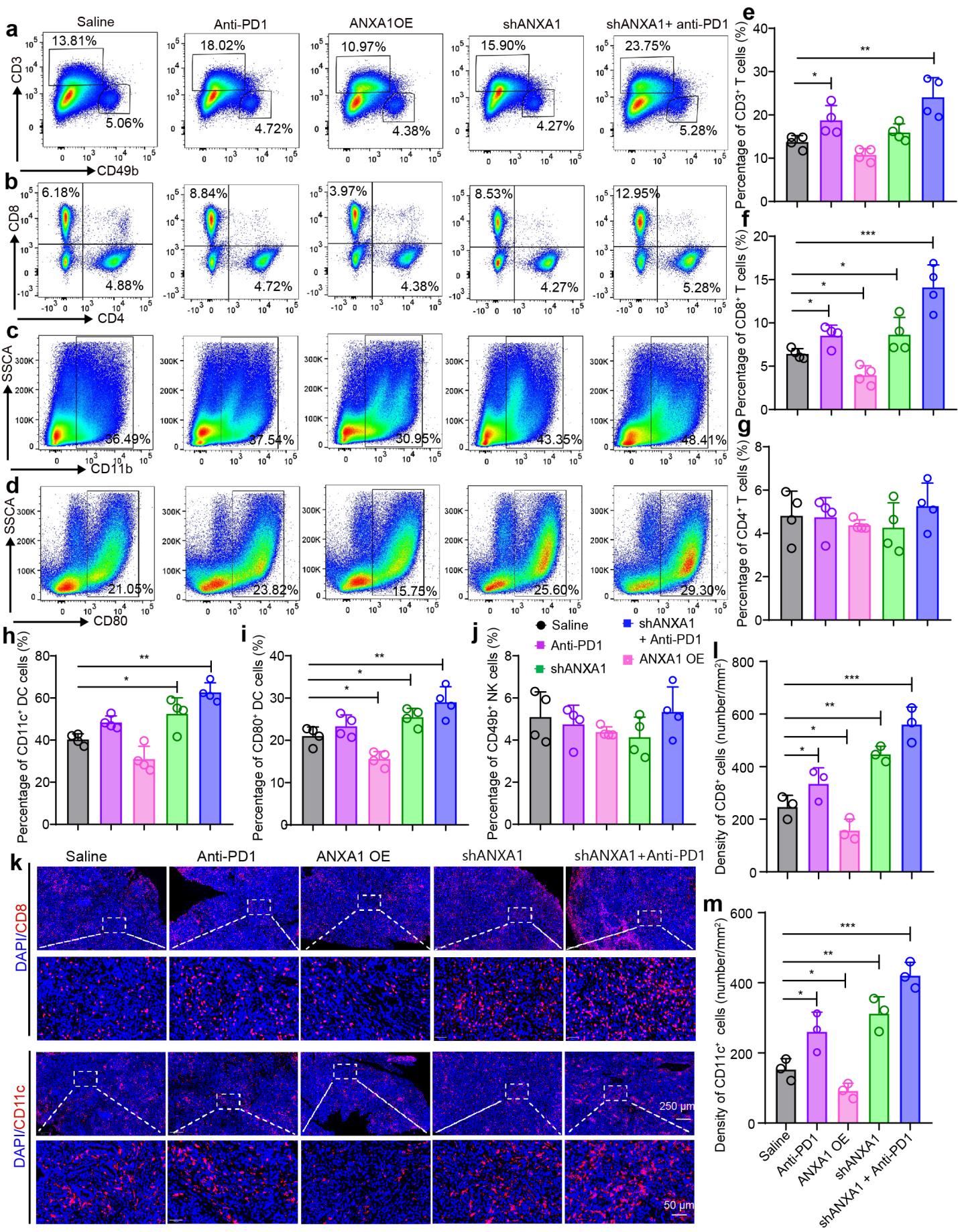


**Supplementary Figure 7. Analysis of tumor-infiltrating immune cells in** **orthotopic PDAC tumors by flow cytometry.**

(a-d) The representative scatter plots of CD3^+^ T cells, CD8^+^ cytotoxic T cells (CTLs, CD3^+^CD8^+^), T helper cells (CD3^+^CD4^+^), total DCs (CD45^+^CD11c^+^), mature DCs (CD11c^+^CD80^+^), and NKs (CD3^-^CD49b^+^) in mouse PDAC orthotopic tumors were presented.

(e-j) The percentage of CD3^+^ T cells (e), CD8^+^ cytotoxic T cells (f), T helper cells (g), total DCs (h), mature DCs (i), and NKs (j) within total CD45^+^ immunocytes of each condition were quantified by flow cytometer. The quantitative results were presented as mean ± SD, *P<0.05, **P<0.01, and ***P<0.001.

(k) Whole slide immunofluorescence staining of CD8 and CD11c to analyze the infiltration of T cells and DCs in the orthotopic mouse PDAC tumors. Scale bar 250 μm or 50 μm.

(l, m) The densities of CD8^+^ T cells (l) and CD11c^+^ DCs (m) in each condition were quantified.


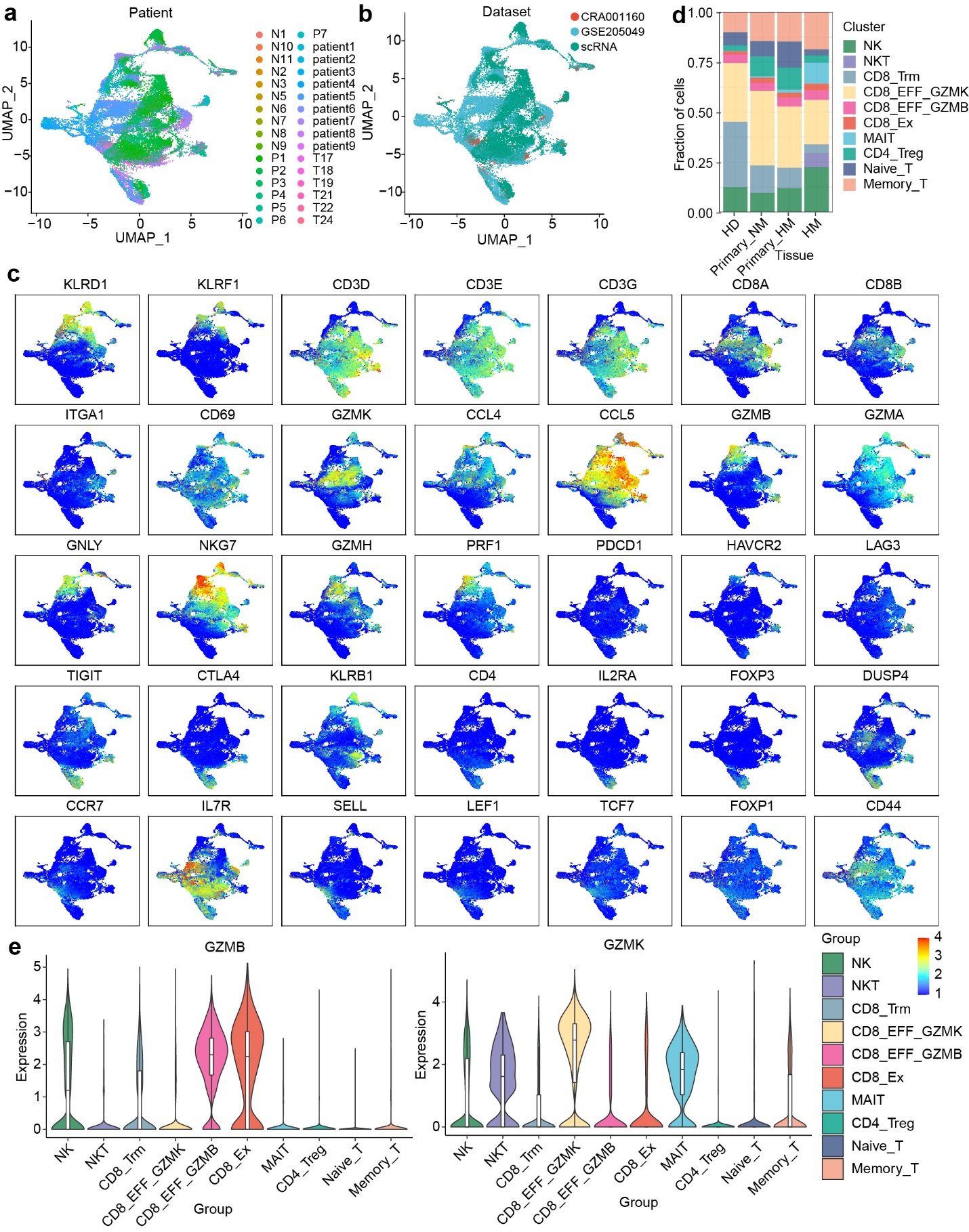


**Supplementary Figure 8. T lymphocyte and NK cell subtype distribution**

(a) A UMAP plot illustrates the sub-clustered T lymphocytes and NK cells from all biopsies, with cells color-coded according to patient origin.

(b) A UMAP plot displays the sub-clustered T lymphocytes and NK cells, color-coded based on dataset origin, indicating the distribution across different studies.

(c) The UMAP plot projects the expression of marker genes for the indicated cell subtypes, ranging from blue to red.

(d) A bar plot quantifies the proportion of T cell subtypes and NK cells within the cohorts HD, Primary_NM, Primary_HM, and HM lesions, providing insights into their relative frequencies.

(e) Violin plots illustrate the GZMK and GZMB expression levels for each cell subtype.


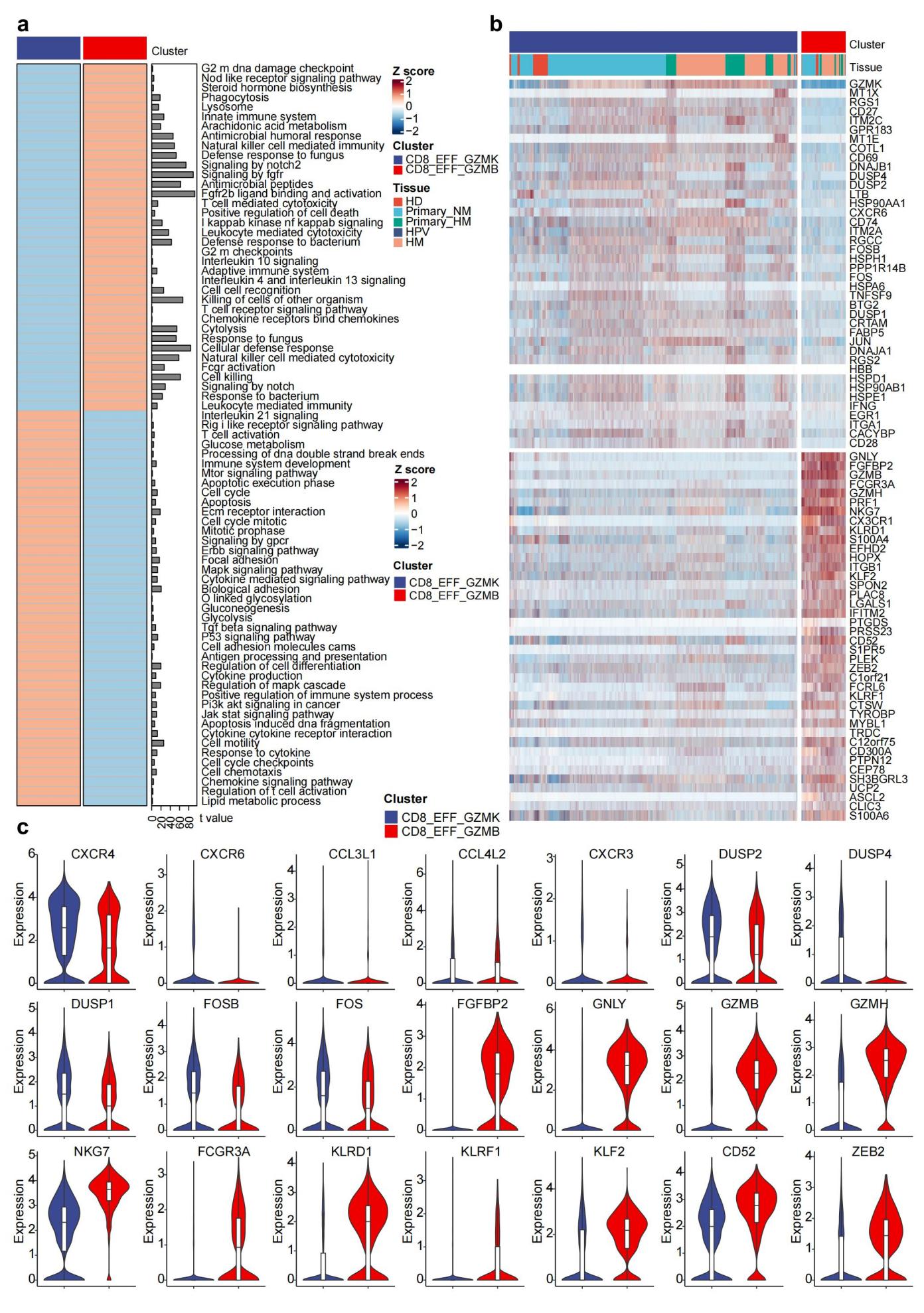


**Supplementary Figure 9. Gene set enrichment and differential expression in CD8 effector T cells**

(a) A heatmap shows the differentially enriched gene sets between CD8^+^EFF GZMK and CD8^+^EFF GZMB cells, with the bar plot on the right representing the t values.

(b) The top 30 differentially expressed genes among CD8^+^EFF GZMK and CD8^+^EFF GZMB cells are presented, with colors representing the normalized z-scores.

(c) Violin plots show the marker genes expression level in CD8^+^EFF GZMK and CD8^+^EFF GZMB cells.


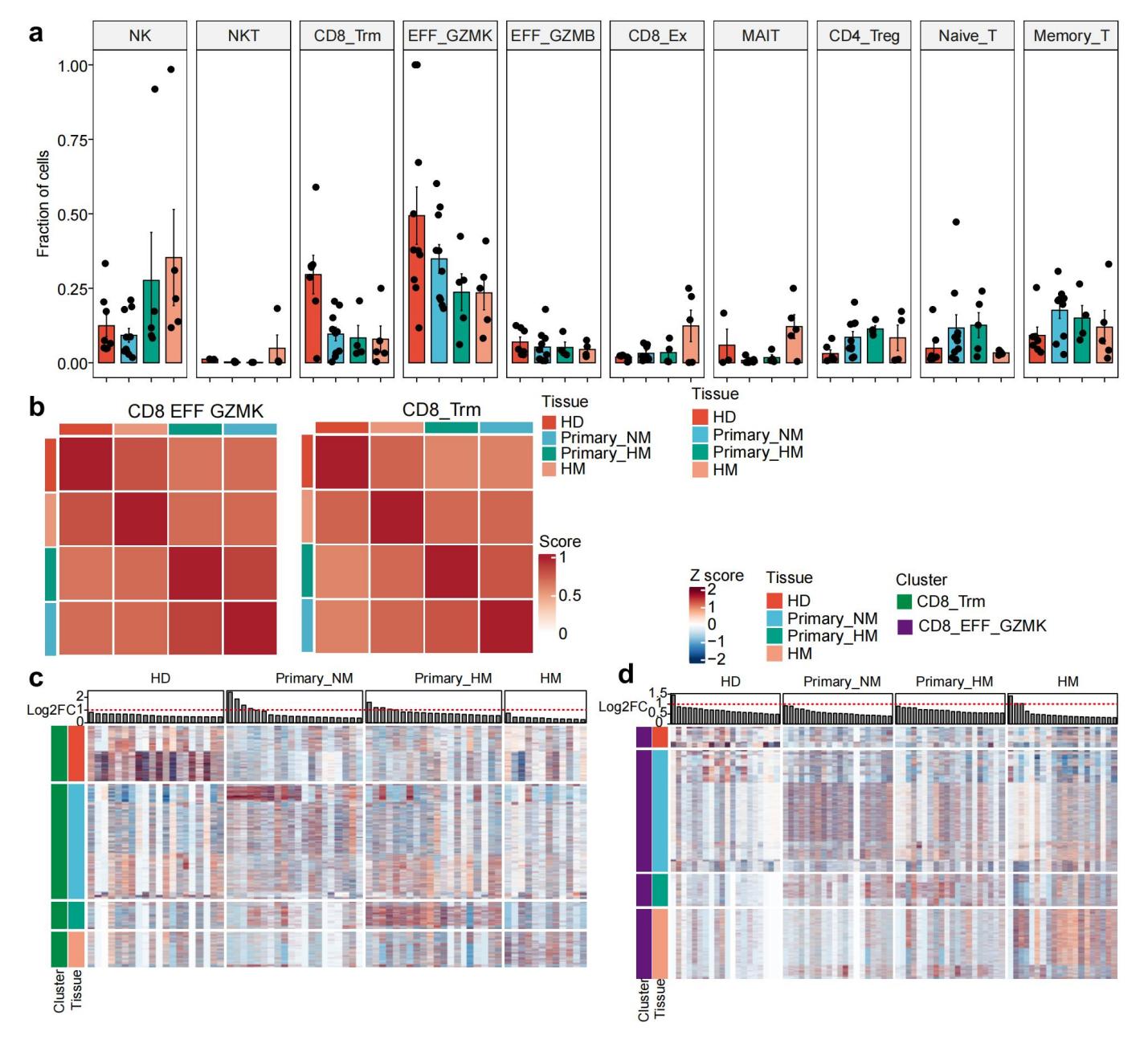


**Supplementary Figure 10. T cell subtype proportions and differential gene expression of CD8 T cells**

1. A bar plot shows the proportion of T cell subtypes and NK cells within the immune cells across all biopsies. Each dot present a sample.

(b) The heatmap represents the correlation of CD8 EFF GZMK cells (left) and Trm cells (right) across different tissues, highlighting the relationships between cell subtypes.

(c) A heatmap illustrates the differential genes of CD8 Trm cells between different tissues, with the bar plot on top representing the Log2 Fold change, indicating the relative expression levels.

(d) A heatmap displays the differential genes of CD8^+^EFF GZMK cells between different tissues, with the bar plot on top representing the Log2 Fold change, highlighting tissue-specific gene expression patterns.


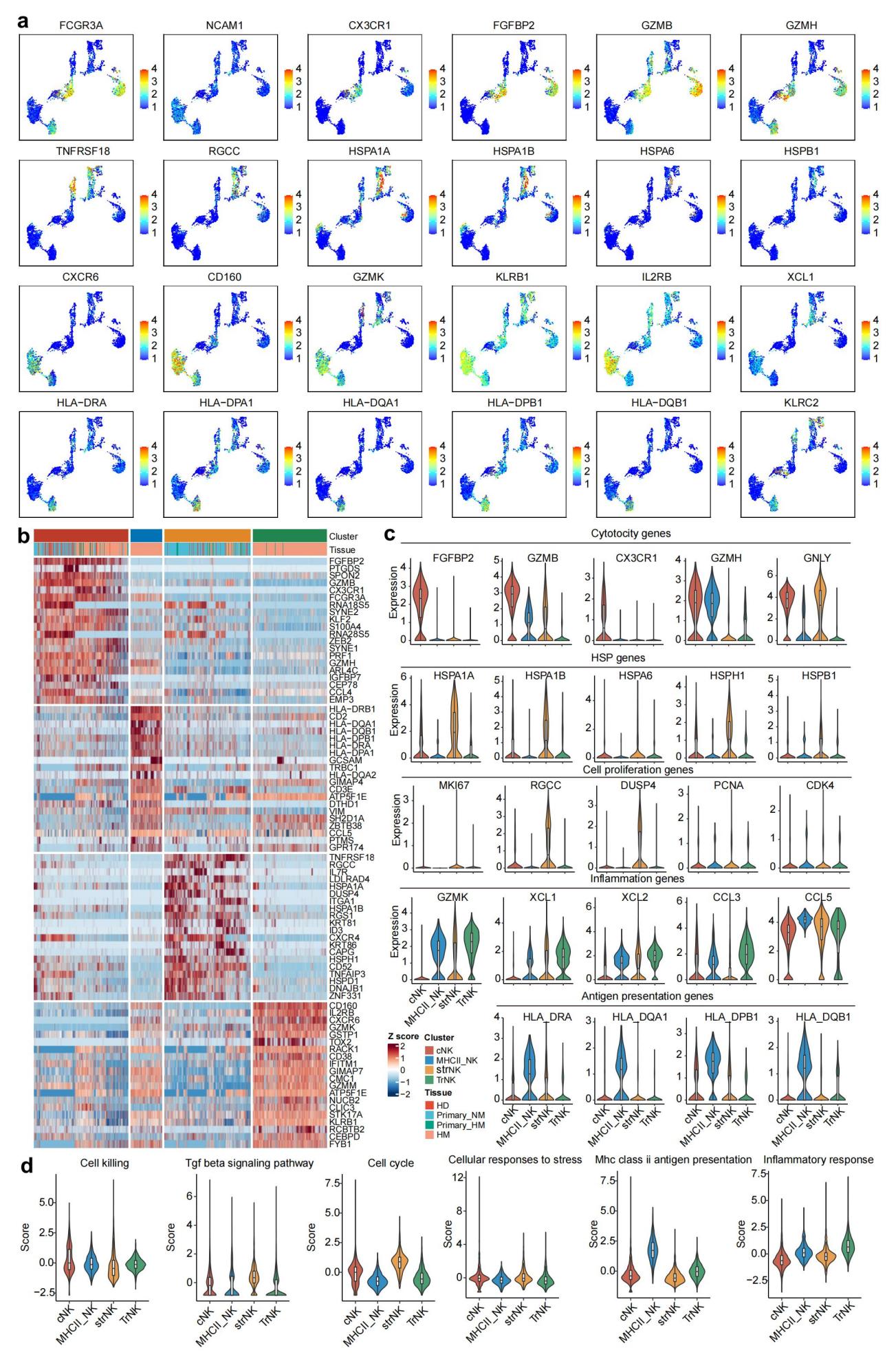


**Supplementary Figure 11. NK cell subtype gene expression and functional profiling**

(a) The UMAP plot projects the expression of marker genes for the indicated NK subtypes, color-coded from blue to red.

(b) A heatmap shows the top 15 DEGs for each NK subtype, with colors representing the normalized z-scores.

(c) Violin plots illustrate the expression of genes related to cytotoxicity, HSP, cell proliferation, inflammation, and antigen presentation for each NK subtype.

(d) Violin plots display the enrichment score of the canonical gene set for the indicated cell subtypes.
